# Supplementary material for: The Isolation and Structure Elucidation of Spirotetronate Lobophorins A, B, and H8 from Streptomyces sp. CB09030 and Their Biosynthetic Gene Cluster
Source: Molecules. 2023 Apr 20;28(8):3597. doi: 10.3390/molecules28083597 (PMC10143065; doi:10.3390/molecules28083597)
Supplement: Supplementary file 1 [file molecules-28-03597-s001.zip › molecules-2273782-supplementary.pdf]

Supplementary material

Table of Contents

Table S1 The comparison of NMR data of compound **1** 4

Table S2 The comparison of NMR data of compound **2** 6

Table S3 The comparison of NMR data of compound **3** 8

Table S4 The comparison of NMR data of compound **4** 9

Table S5 AntiSMASH-predicted BGCs for *S*. sp. CB09030. 11

Table S6 Deduced function of open reading frames (ORFs) in the *lob* BGC. 12

Table S7 The antibacterial activities of LOBs. 14

Figure S1 Anti-mycobacterial bioassay of the individual fermentation products. 15

Figure S2 The purified compounds **1-4**. 16

Figure S3 AntiSMASH-predicted BGCs of putative natural products encoded in the genome of *S*. sp. CB09030. 17

Figure S4 Sequence analysis of glycosyltransferases from three *Streptomyces* spp. 18

Figure S5 Antibacterial assays of compounds **1** – **4** against *M. smegmatis* MC^2 155 and *B. subtilis* 62305 using broth dilution assay. 19

Figure S6 The cytotoxicity of compounds **1** 19

Figure S7 The HMRS and MS/MS of compound **1** 20

Figure S8 The MS/MS of compound **2** 21

Figure S9 The HRMS of compound **3** 22

Figure S10 The HRMS of compound **4** 22

Figure S11 ^{1}H-NMR spectrum of **1** in CD_3OD (400 MHz) 23

Figure S12 ^{13}C NMR spectrum of **1** in CD_3OD (100 MHz) 24

Figure S13 ^{1}H NMR spectrum of **2** in CD_3OD (600 MHz). 25

Figure S14 ^{13}C NMR spectrum of **2** in CD_3OD (150 MHz) 26

Figure S15 DEPT 90 spectrum of **2** in CD_3OD 27

Figure S16 DEPT 135 spectrum of **2** in CD_3OD 27

Figure S17 ^{1}H-^{1}H COSY spectrum of **2** in CD_3OD 28

Figure S18 HSQC spectrum of **2** in CD_3OD 28

Figure S19 HMBC spectrum of **2** in CD_3OD 29

Figure S20 NOESY spectrum of **2** in CD_3OD 29

Figure S21 ^{1}H NMR spectrum of **3** in CD_3OD (500 MHz) 30
Figure S22 ^{13}C-NMR spectrum of **3** in CD_3OD (125 MHz) 31
Figure S23 DEPT 90 spectrum of **3** in CD_3OD 32
Figure S24 DEPT 135 spectrum of **3** in CD_3OD 32
Figure S25 ^{1}H-^{1}H COSY spectrum of **3** in CD_3OD 33
Figure S26 HSQC spectrum of **3** in CD_3OD 33
Figure S27 HMBC spectrum of **3** in CD_3OD 34
Figure S28 NOESY spectrum of **3** in CD_3OD 34
Figure S29 ^{1}H NMR spectrum of **4** in CD_3OD (600 MHz) 35
Figure S30 ^{13}C NMR spectrum of **4** in CD_3OD (150 MHz) 36
Figure S31 DEPT 90 spectrum of **4** in CD_3OD 37
Figure S32 DEPT 135 spectrum of **4** in CD_3OD 37
Figure S33 ^{1}H-^{1}H COSY spectrum of **4** in CD_3OD 38
Figure S34 HSQC spectrum of **4** in CD_3OD 38
Figure S35 HMBC spectrum of **4** in CD_3OD 39
Figure S36 NOESY spectrum of **4** in CD_3OD 39

Table S1 The comparison of NMR data of compound **1**.

**Position**	**1**[a]		**LOB A**[b] [1]
	δ_C	δ_H	δ_C
	type	(*J* in Hz)	
**1**	177.7, C		173.5
**2**	99.8, C		100.0
**3**	203.0, C		201.3
**4**	52.6, C		51.7
**5**	45.6, CH	2.11 (m, o[c])	44.6
**6**	36.6, CH	1.63 (m, o)	31.6
**7**	43.3, CH_2	1.59, 1.50 (m, o)	42.6
**8**	36.6, CH	2.26 (m, o)	35.1
**9**	86.6, CH	3.43 (m, o)	84.6
**10**	40.4, CH	2.05 (m, o)	39.3
**11**	126.7, CH	5.73 (d, 10.4)	126.3
**12**	129.3, CH	5.38 (m, o)	128.3
**13**	56.0, CH	3.57 (m, o)	51.7
**14**	137.7, C		137.2
**15**	122.5, CH	5.22 (d, 9.2)	123.0
**16**	32.6, CH_2	2.42, 2.23 (m, o)	31.9
**17**	80.8, CH	4.25, s	79.8
**18**	137.6, C		136.0
**19**	121.5, CH	5.12, (d, 10.1)	121.6
**20**	41.5, CH	3.44 (m, o)	41.2
**21**	124.0, CH	5.41, s	123.9
**22**	141.7, C		142.5
**23**	29.3, CH	2.50 (m, o)	28.3
**24**	36.6, CH_2	2.31, 1.63 (m, o)	36.0
**25**	85.1, C		83.7
**26**	200.3, C		200.9
**27**	15.7, CH_3	1.50, s	15.5
**28**	23.2, CH_3	0.67 (d, 5.5)	22.9
**29**	14.9, CH_3	1.14 (d, 6.7)	14.6
**30**	15.5, CH_3	1.41, s	14.6
**31**	14.8, CH_3	1.38, s	14.9
**32**	65.3, CH_2	4.16, 4.09 (m, o)	65.0
**33**	20.7, CH_3	1.30 (d, 6.9)	20.2
$\mathbf{1_A}$	99.4, CH	4.77 (d, 3.7)	98.4
$\mathbf{2_A}$	30.3, CH_2	2.39, 1.73 (m, o)	30.3
$\mathbf{3_A}$	68.8, CH	4.04 (m, o)	67.2
$\mathbf{4_A}$	73.4, CH	3.31 (m, o)	72.3
$\mathbf{5_A}$	65.3, CH	4.15 (m, o)	65.4

**6_A**	18.6, CH_3	1.26 (m, o)	18.6
**1_B**	93.2, CH	5.19 (d, 5.31)	92.0
**2_B**	35.9, CH_2	2.21, 2.01 (m, o)	36.0
**3_B**	68.0, CH	4.20 (m, o)	67.2
**4_B**	83.8, CH	3.31 (m, o)	82.6
**5_B**	63.6, CH	4.07 (m, o)	63.0
**6_B**	18.2, CH_3	1.24 (m, o)	17.9
**1_C**	100.7, CH	4.97 (m, o)	100.0
**2_C**	38.8, CH_2	2.06, 1.74 (m, o)	38.7
**3_C**	64.4, CH	4.31, s	63.2
**4_C**	83.8, CH	2.87 (dd, 9.6, 2.9)	83.5
**5_C**	69.3, CH	3.97 (m, o)	68.6
**6_C**	18.3, CH_3	1.22 (m, o)	18.6
**7_C**	57.6, CH_3	3.40, s	56.3
**1_D**	97.9, CH	4.73 (dd, 9.7, 1.6)	98.4
**2_D**	38.0, CH_2	2.07, 1.81 (m, o)	36.0
**3_D**	57.5, C		91.6[d]
**4_D**	55.8, CH	3.78 (m, o)	54.7
**5_D**	69.7, CH	3.82 (m, o)	69.1
**6_D**	17.4, CH_3	1.18 (d, 6.2)	17.3
**7_D**	29.5, CH_3	1.33, s	25.3
**8_D**	160.0, C		158.8
**9_D**	52.7, CH_3	3.70, s	52.3

[a] Recorded in CD_3OD at 600 MHz for ^1H NMR, 150 MHz for ^{13}C NMR. [b] Recorded in pyridine-d_5 for ^{13}C NMR. [c] Overlapped. [d] This position connects the amino group and the chemical shift should be around 50 ppm.

Table S2 The comparison of NMR data of compound **2**.

**Position**	**2**[a]		**LOB B**[b] [2]
	δ_C	δ_H	δ_C
	type	(J in Hz)	type
**1**	170.3, C		170,1
**2**	103.4, C		100.3
**3**	205.7, C		206.0
**4**	52.5, C		52.5
**5**	44.7, CH	2.10 (m, o[C])	44.9
**6**	32.4, CH	1.63 (m, o)	32.6
**7**	42.9, CH_2	1.63, 1.56 (m, o)	43.1
**8**	35.9, CH	2.26 (m, o)	36.1
**9**	85.9, CH	3.45 (m, o)	85.9
**10**	39.7, CH	2.11 (m, o)	39.7
**11**	127.6, CH	5.85 (d, 10.1)	127.6
**12**	127.4, CH	5.40 (m, o)	127.7
**13**	54.2, CH	3.55 (m, o)	54.5
**14**	137.2, C		137.3
**15**	124.8, CH	5.21 (d, 9.9)	125.1
**16**	32.1, CH_2	2.44, 2.28 (m, o)	32.1
**17**	80.1, CH	4.28, s	80.2
**18**	139.1, C		139.3
**19**	120.2, CH	5.19 (m, o)	120.4
**20**	41.6, CH	3.66 (d, 11.0)	41.7
**21**	123.5, CH	5.56, s	123.6
**22**	142.7, C		143.0
**23**	28.5, CH	2.77 (t, 6.6)	28.7
**24**	36.2, CH_2	2.41, 1.81 (m, o)	36.3
**25**	84.9, C		84.9
**26**	200.4, C		200.5
**27**	15.5, CH_3	1.60, s	15.5
**28**	22.9, CH_3	0.68, s	22.9
**29**	14.8, CH_3	1.16 (m, o)	14.8
**30**	14.1, CH_3	1.41, s	15.2
**31**	15.2, CH_3	1.45, s	14.2
**32**	65.2, CH_2	4.22, 4.07 (m, o)	65.4
**33**	20.3, CH_3	1.33 (d, 7.2)	20.3
**1_A**	99.7, CH	4.77, s	99.8
**2_A**	31.0, CH_2	2.37, 1.76 (d, 14.8)	31.2
**3_A**	69.2, CH	4.04, s	69.5
**4_A**	73.2, CH	3.32 (m, o)	73.5
**5_A**	65.9, CH	4.12 (m, o)	66.2
**6_A**	18.2, CH_3	1.23 (m, o)	17.3

**1_B**	93.1, CH	5.17 (m, o)	93.3
**2_B**	35.9, CH_2	2.09, 2.01 (m, o)	36.5
**3_B**	68.0, CH	4.21 (m, o)	69.2
**4_B**	83.3, CH	3.31 (m, o)	83.3
**5_B**	63.6, CH	4.10 (m, o)	63.7
**6_B**	18.0, CH_3	1.23 (m, o)	18.3
**1_C**	100.7, CH	4.96 (d, 9.7)	100.9
**2_C**	38.8, CH_2	2.07, 1.73 (m, o)	39.0
**3_C**	64.4, CH	4.28, s	64.4
**4_C**	83.7, CH	2.88 (d, 9.2)	83.8
**5_C**	69.6, CH	3.82 (m, o)	69.8
**6_C**	18.7, CH_3	1.23 (m, o)	18.8
**7_C**	57.0, CH_3	3.40, s	57.0
**1_D**	99.0, CH	4.51 (d, 9.3)	99.1
**2_D**	36.3, CH_2	2.68, 1.82 (m, o)	38.7
**3_D**	92.3, C		92.6
**4_D**	55.2, CH	4.38 (m, o)	55.3
**5_D**	70.0, CH	3.54 (m, o)	70.5
**6_D**	17.0, CH_3	1.16 (m, o)	14.1
**7_D**	25.8, CH_3	1.55, s	26.0
**8_D**	160.0, C		160.2
**9_D**	52.9, CH_3	3.72, s	53.1
**D-4NH**		7.25 (d, 10.0)	

[a] Recorded in CD_3OD at 600 MHz for ^1H NMR, 150 MHz for ^{13}C NMR. [b] Recorded in CD_3OD at 150 MHz for ^{13}C NMR. [c] Overlapped.

Table S3 The comparison of NMR data of compound **3**.

**Position**	**3**[a]		**32-chloro-32-deoxykijanolide**[b] [3]
	δ_C	δ_H	δ_C
	type	(J in Hz)	type
**1**	170.2, C		170.3
**2**	103.7, C		103.3
**3**	206.2, C		206.1
**4**	52.5, C		52.4
**5**	44.5, CH	2.06 (m, o[c])	32.4
**6**	32.1, CH	1.63 (m, o)	28.7
**7**	43.1, CH_2	1.57 (m, o)	43.1
**8**	36.5, CH	2.19 (m, o)	40.2
**9**	76.8, CH	3.59 (m, o)	76.8
**10**	40.4, CH	2.08 (m, o)	36.2
**11**	127.9, CH	6.07 (d, 10.2)	127.3
**12**	127.3, CH	5.39 (ddd, 10.2, 4.8, 2.1)	127.9
**13**	54.5, CH	3.55 (d, 4.7)	54.3
**14**	136.9, C		136.9
**15**	124.8, CH	5.27(t, 9.0)	124.5
**16**	36.3, CH_2	2.30, 1.81 (m, o)	33.0
**17**	73.6, CH	4.15, s	73.5
**18**	141.8, C		139.3
**19**	119.5, CH	5.27 (m, o)	127.3
**20**	41.7, CH	3.59 (m, o)	44.4
**21**	122.1, CH	5.18, s	118.4
**22**	138.7, C		142.5
**23**	36.3, CH	2.39 (d, 12.2)	41.7
**24**	33.0, CH_2	2.41, 2.14 (m, o)	36.2
**25**	84.7, C		84.4
**26**	200.7, C		200.8
**27**	15.6, CH_3	1.60, s	15.3
**28**	22.9, CH_3	0.67 (d, 5.6)	23
**29**	13.6, CH_3	1.04, (d, 7.1)	13.7
**30**	14.1, CH_3	1.42, s	15.7
**31**	15.1, CH_3	1.41, s	14.2
**32**	22.1, CH_3	1.81, s	-[d]
**33**	20.6, CH_3	1.28 (d, 7.2)	20.1

[a] Recorded in CD_3OD at 500 MHz for ^1H NMR, 125 MHz for ^{13}C NMR. [b] Recorded in CD_3OD for ^{13}C NMR. [c] Overlapped. [d] Obscured by CD_3OD signal. 32-chloro-32-deoxykijanolide is replaced by chloromethyl at the 32nd position of **3**. LOB H8 was dissolved in DMSO-d_6, so the data were not compared.

Table S4 The comparison of NMR data of compound **4**.

**Position**	**4**[a]		***O*-β-D-kijanosyl-(1→17)-kijanolide**[b] [3]
	**δ_C**	**δ_H**	**δ_C**
	type	(J in Hz)	type
**1**	171.3, C		167.1
**2**	102.5, C		102.0
**3**	205.0, C		206.5
**4**	52.6, C		51.1
**5**	40.4, CH	2.02 (m, o[c])	31.2
**6**	32.6, CH	1.56 (m, o)	28.0
**7**	43.2, CH_2	1.56, 1.50 (m, o)	41.9
**8**	36.4, CH	2.16 (m, o)	39.3
**9**	77.1, CH	3.54 (m, o)	76.1
**10**	44.7, CH	2.02 (m, o)	34.8
**11**	124.5, CH	6.0 (d, 10.1)	125.8
**12**	124, CH	5.32 (dd, 10.0, 4.6)	126.5
**13**	54.1, CH	3.49 (m, o)	53.3
**14**	137.7, C		135.9
**15**	124.5, CH	5.16 (d, 9.8)	123.4
**16**	32.3, CH_2	2.40, 2.21 (m, o)	31.2
**17**	80.5, CH	4.20 (m, o)	78.6
**18**	139.3, C		137.0
**19**	120.5, CH	5.12 (d, 10.5)	121.5
**20**	41.7, CH	3.67 (m, o)	42.9
**21**	124, CH	5.48, s	119.4
**22**	142.7, C		141.5
**23**	28.7, CH	2.68 (t, 6.9)	40.3
**24**	36.4, CH_2	2.35, 1.78 (m, o)	35.4
**25**	85.1, C		83.3
**26**	200.7, C		201.5
**27**	15.7, CH_3	1.53, s	15.0
**28**	23.2, CH_3	0.61 (d, 4.2)	22.3
**29**	13.8, CH_3	0.99 (d, 7.1)	13.0
**30**	14.3, CH_3	1.35, s	15.2
**31**	15.4, CH_3	1.38, s	13.7
**32**	65.4, CH_2	4.17, 4.01 (d, 12.8)	64.9
**33**	20.5, CH_3	1.27 (d, 7.3)	20.2
**1_D**	99.2, CH	4.47 (d, 9.6)	97.1
**2_D**	36.6, CH_2	2.62 (d, 14.3)	35.8
**3_D**	92.5, C	1.80 (m, o)	91.2
**4_D**	55.4, CH		53.8
**5_D**	70.4, CH	4.31 (d, 9.7)	69.2

**6_D**	17.4, CH_3	3.48 (m, o)	17.0
**7_D**	26.0, CH_3	1.09 (d, 6.1)	25.3
**8_D**	160.3, C	1.49, s	157.4
**9_D**	53.2, CH_3		52.6
**D-4NH**		3.65, s	

[a] Recorded in CD_3OD at 400 MHz for ^1H NMR, 101 MHz for ^{13}C NMR. [b] Recorded in $CDCl_3$ for ^{13}C NMR. [c] overlapped.

Table S5 AntiSMASH-predicted BGCs for *S.* sp. CB09030.

Region	Type	Position		Product
		From	To	
Region 1	Lanthipeptide-class II	307,397	335,569	SBI-06990 A1, SBI-06990 A2
Region 2	NRPS	1,236,052	1,289,155	ishigamide
Region 3	Betalactone	1,406,082	1,431,639	julichrome Q3-3, julichrome Q3-5
Region 4	Terpene	1,447,488	1,466,387	albaflavenone
Region 5	T2PKS	1,529,493	1,598,257	spore pigment
Region 6	Siderophore	2,067,208	2,077,382	\
Region 7	Lanthipeptide-class-i	2,159,332	2,181,980	SapB
Region 8	T2PKS, PKS-like, NRPS	2,208,262	2,294,786	olimycin A, olimycin B
Region 9	NRPS, T1PKS	2,354,409	2,401,914	xiamycin A
Region 10	RiPP-like	2,437,839	2,449,111	cyclothiazomycin
Region 11	Terpene	2,478,990	2,500,577	geosmin
Region 12	Siderophore	2,657,445	2,670,619	\
Region 13	NRPS	2,714,868	2,758,137	diisonitrile antibiotic SF2768
Region 14	NRPS, Nucleoside	2,809,938	2,852,748	nogalamycin
Region 15	Terpene, NRPS	3,162,527	3,235,065	hopene
Region 16	NRPS, T1PKS, NRPS-like	3,283,414	3,409,750	divergolide A-D
Region 17	NRPS	3,455,294	3,518,212	cyclomarin D
Region 18	Terpene	3,785,165	3,803,571	versipelostatin
Region 19	RiPP-like	3,815,011	3,825,226	informatipeptin
Region 20	NRPS	4,046,857	4,096,234	coelichelin
Region 21	T1PKS	4,222,195	4,308,855	elaiophylin
**Region 22**	**T1PKS, NRPS**	**4,329,164**	**4,544,509**	**lobophorin A**
Region 23	Terpene	4,562,586	4,582,407	ebelactone
Region 24	T3PKS	4,615,541	4,656,093	germicidin
Region 25	Indole	4,802,524	4,823,660	5-isoprenylindole-3-carboxylate β-D-glycosyl ester
Region 26	Terpene	4,876,326	4,900,561	carotenoid
Region 27	Amglyccycl	5,152,346	5,173,584	β-D-galactosylvalidoxylamine-A
Region 28	T3PKS	5,387,878	5,428,990	herboxidiene
Region 29	NRPS	5,500,600	5,554,020	rimosamide
Region 30	Ectoine	6,078,187	6,088,585	ectoine
Region 31	Melanin	7,076,380	7,087,006	istamycin
Region 32	Lassopeptide	7,146,748	7,169,269	SSV-2083
Region 33	Lanthipeptide-class-i	7,717,072	7,743,529	\
Region 34	Phenazine	7,926,118	7,946,558	\

Table S6 Deduced function of open reading frames (ORFs) in the *lob* BGC.

ORF	Size (aa)	Proposed Function	*Streptomyces* sp. SCSIO 01127		*Streptomyces olivaceus* SCSIO T05		*Streptomyces* sp. FXJ 7.023	
			ID/SI (%)	Protein Homologue	ID/SI (%)	Protein Homologue	ID/SI (%)	Protein Homologue
Orf (-2)	393	macrolide glycosyltransferase	98/98	AGI99472.1	98/98	QFU80876.1	98/98	AGC09509.1
Orf (-1)	260	FkbM family methyltransferase	97/98	AGI99473.1	97/98	QFU80877.1	97/98	AGC09508.1
lobR1	195	TetR type regulatory protein	96/96	AGI99474.1	96/96	QFU80878.1	95/94	AGC09507.1
lobT1	497	efflux permease	99/99	AGI99475.1	99/99	QFU80879.1	99/99	AGC09506.1
lobP1	392	p450 monooxygenase	98/99	AGI99476.1	98/99	QFU80880.1	98/98	AGC09505.1
lobU1	326	aldo/keto reductase	99/100	AGI99477.1	99/100	QFU80881.1	99/100	AGC09504.1
lobS1	272	sugar-O-methyltransferase	99/100	AGI99478.1	99/100	QFU80882.1	100/100	AGC09503.1
lobS2	384	sugar 4-aminotransferase	98/98	AGI99479.1	98/98	QFU80883.1	98/98	AGC09502.1
lobS3	266	SAM-dependent methyltransferase	99/99	AGI99480.1	99/98	QFU80884.1	99/99	AGC09501.1
lobG1	391	glycosyltransferase	99/99	AGI99481.1	99/99	QFU80886.1	98/98	AGC09500.1
lobA1	3936	TI-PKS	93/93	AGI99482.1	93/93	QFU80887.1	98/98	AGC09499.1
lobS4	483	sugar 2,3-dehydratase	98/98	AGI99483.1	98/98	QFU80888.1	98/98	AGC09498.1
lobB	253	thioesterase	99/99	AGI99484.1	99/99	QFU80889.1	99/100	AGC09497.1
lobP2	506	FAD-dependent oxidoreductase	98/99	AGI99485.1	98/99	QFU80890.1	99/99	AGC09496.1
lobG2	416	glycosyltransferase	99/99	AGI99486.1	99/100	QFU80891.1	99/99	AGC09495.1
lobG3	400	glycosyltransferase	99/99	AGI99487.1	99/100	QFU80892.1	99/99	AGC09494.1
lobR2	274	TetR-type regulatory protein	97/99	AGI99488.1	\	\	99/99	AGC09493.1
lobC1	616	hydrolase superfamily dihydrolipoamide acyltransferase-like protein	97/98	AGI99489.1	99/99	QFU80893.1	97/98	AGC09492.1
lobC2	75	ACP	100/100	AGI99490.1	99/100	QFU80894.1	99/100	AGC09491.1

lobC3	621	FkbH-like protein	98/98	AGI99491.1	99/100	QFU80895.1	98/99	AGC09490.1
lobC4	352	ketoacyl acylcarrier protein synthase III	99/98	AGI99492.1	99/99	QFU80896.1	98/98	AGC09489.1
lobP3	492	FAD-dependent oxidoreductase	98/99	AGI99493.1	98/99	QFU80897.1	98/99	AGC09488.1
lobA2	1590	TI-PKS	94/95	AGI99494.1	99/99	QFU80898.1	95/95	AGC09487.1
lobA3	1815	TI-PKS	84/87	AGI99495.1	98/98	QFU80899.1	83/87	AGC09486.1
lobA4	7449	TI-PKS	95/95	AGI99496.1	99/98<br>97/97	QFU80900.1<br>QFU80901.1	95/95	AGC09485.1
lobA5	6363	TI-PKS	97/97	AGI99497.1	98/98	QFU80902.1	97/97	AGC09484.1
lobU2	133	unknown	97/98	AGI99498.1	100/100	QFU80903.1	98/98	AGC09483.1
lobS5	414	sugar 3-C-methyl transferase	98/99	AGI99499.1	99/99	QFU80904.1	99/99	AGC09482.1
lobS6	373	sugar 3-aminotransferase	98/98	AGI99500.1	99/100	QFU80905.1	98/98	AGC09481.1
lobS7	439	FAD-dependent oxidoreductase	99/99	AGI99501.1	99/100	QFU80906.1	99/99	AGC09480.1
lobS8	344	sugar 4,6-dehydratase	99/99	AGI99502.1	99/99	QFU80907.1	99/99	AGC09479.1
lobS9	298	sugar nucleotidyltransferase	98/99	AGI99503.1	99/100	QFU80908.1	99/99	AGC09478.1
lobS10	332	sugar 3-ketoreductase	99/99	AGI99504.1	99/99	QFU80909.1	99/99	AGC09477.1
lobS11	202	sugar 5-epimerase	99/100	AGI99505.1	99/100	QFU80910.1	99/100	AGC09476.1
lobR3	274	TetR type regulatory protein	99/99	AGI99506.1	99/100	QFU80911.1	99/99	AGC09475.1
lobT2	128	nuclear transport factor 2 family protein	99/98	AGI99507.1	99/100	QFU80912.1	/	/
lobR4	134	reductase/alcohol dehydrogenase	98/99	AGI99508.1	99/100	QFU80913.1	100/100	AGC09473.1
lobR5	975	reductase/alcohol dehydrogenase	99/100	AGI99509.1	99/100	QFU80914.1	100/100	AGC09472.1
orf1	385	acetyltransferase	100/100	AGI99510.1	100/100	QFU80915.1	99/100	AGC09471.1

Table S7 The antibacterial activities of LOBs.

The antibacterial activities of LOBs (MIC, μg/mL)					
Compounds	*M. tuberculosis*	*B. subtilis*	*S. aureus* ATCC 29213	*B. thuringensis* SCSIO BT01	References
LOB A	32	12.5/0.5/16	>128	16	[2] [4] [5]
LOB B	16	1.56/0.25/0.5	64	2	[2] [4] [5]
LOB C	-	-	-	-	[6]
LOB D	-	-	-	-	[6]
LOB E	-	0.25	32/16	-	[7] [13]
LOB F	-	-	8	-	[7]
LOB G	32	3.125	>50	-	[2]
LOB H	-	1.57	50	-	[8]
LOB I	-	50	>100	-	[8]
LOB J	-	-	-	-	[9]
LOB K	-	-	-	-	[10]
LOB L	-	-	>128	-	[4]
LOB CR1	-	-	-	-	[11]
LOB CR2	-	-	-	-	[11]
LOB CR3	-	-	-	-	[11]
LOB CR4	-	8	64	-	[11] [12]
LOB H1	-	> 64	> 64	-	[5]
LOB H2	-	> 64	> 64	-	[5]
LOB H3	-	> 64	64	-	[5]
LOB H4	-	32	32	-	[5]
LOB H5	-	> 64	> 64	-	[5]
LOB H6	-	> 64	> 64	-	[5]
LOB H7	-	4	4	-	[5]
LOB H8	-	> 64	64	-	[5]
LOB H9	-	16	32	-	[5]
LOB H10	-	> 64	> 64	-	[5]
LOB H11	-	32	32	-	[5]
LOB H12	-	8	8	-	[5]
LOB H13	-	> 64	> 64	-	[5]
LOB H14	-	64	64	-	[5]
LOB H15	-	16	> 64	-	[5]
LOB N1	-	0.5	32	-	[13]
LOB N2	-	1	> 64	-	[13]
LOB N3	-	0.5	> 64	-	[13]

-: No cytotoxicity observed or no test

Figure S1 Anti-mycobacterial bioassay of the individual fermentation products. (A) The activity was measured by paper disk assay on the 27th day; *Streptomyces* strains that produced a bacteriostatic zone were circled in red. (B) HPLC analyses of the fermentation products. (i) blank control medium; (ii) *S*. sp. CB09030 fermentation medium; (iii) *S*. sp. CB00657 fermentation medium; (iv) *S*. sp. CB01580 fermentation medium; (v) *S*. sp. CB02366 fermentation medium.

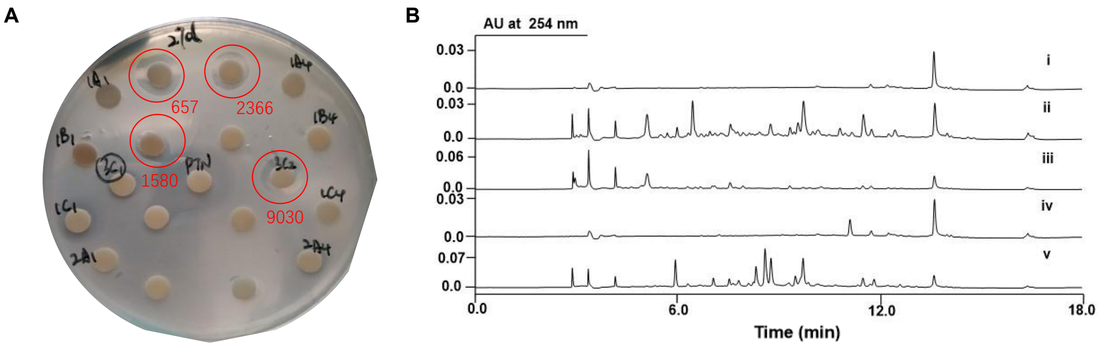

Figure S2 The purified compounds **1–4**. (A) HPLC analyses of the crude extract and purified **1–3**. " x " refers to residual peaks from the chromatographic column. (B) UPLC analyses and HRMS of **4** from the hydrolysis of **2**.

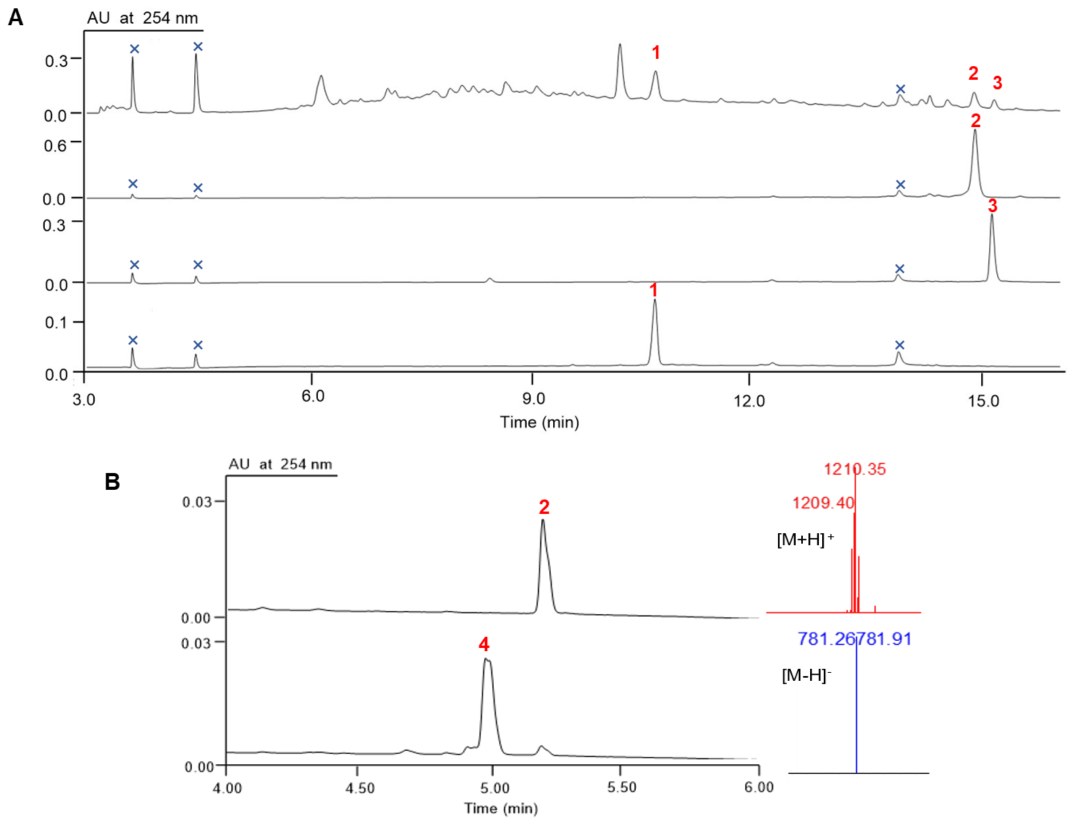

Figure S3 AntiSMASH-predicted BGCs of putative natural products encoded in the genome of *S*. sp. CB09030.

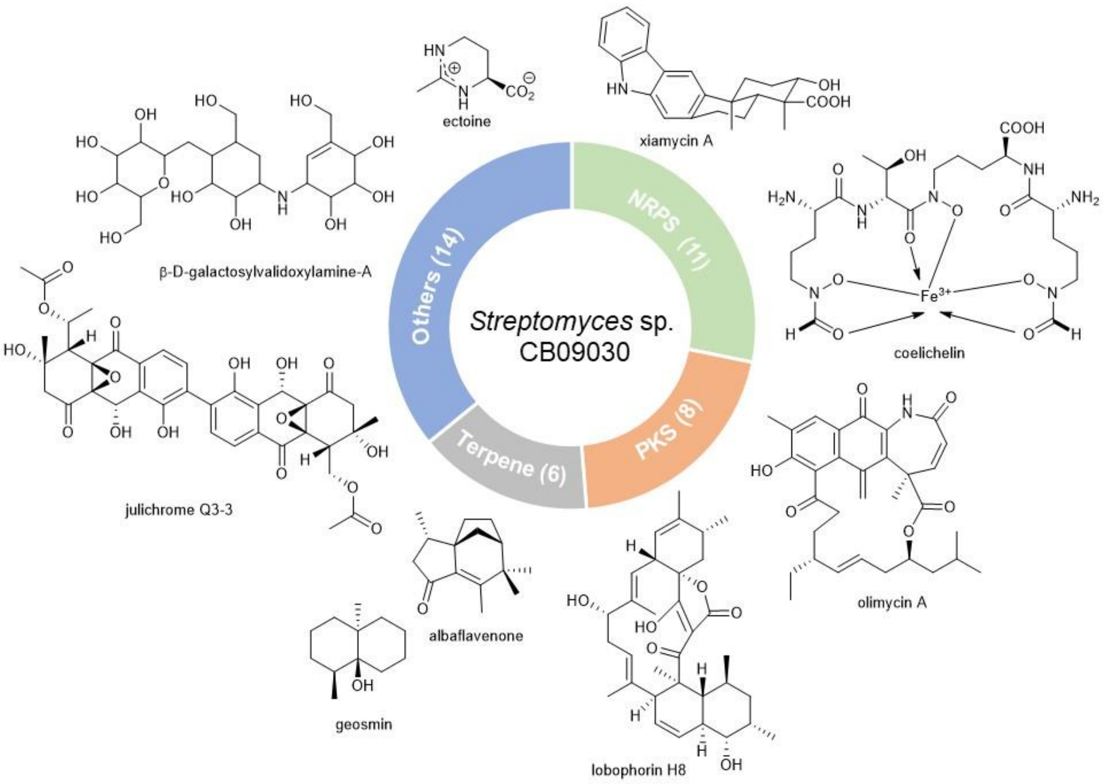

Figure S4 Sequence analysis of glycosyltransferases from three *Streptomyces* spp. (**A**) Phylogenetic tree of LobG1 from *S.* sp. CB09030, LobG1 (AGI99481.1) from *S.* sp. SCSIO 01127 and LobG1 (QFU80886.1) from *S. olivaceus* SCSIO T05. LobG2 (AGI99486.1) and LobG3 (AGI99487.1) from *S.* sp. SCSIO 01127; LobG2 (QFU80891.1) and LobG3 (QFU80892.1) from *S. olivaceus* SCSIO T05. Phylogenetic trees were constructed by MEGA 7.0 software. (**B**) Organization of the LOB biosynthetic gene cluster in three *Streptomyces* strains. Glycosyltransferases were highlighted in red and type I PKS were drew disproportionately in light grey.

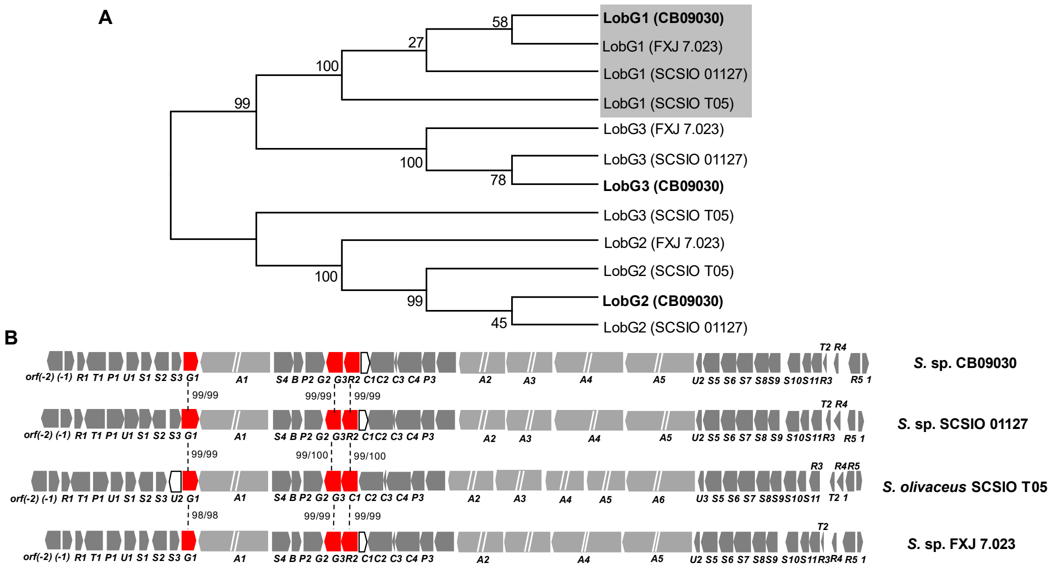

Figure S5 Antibacterial assays of compounds **1 – 4** against *M. smegmatis* MC[2] 155 and *B. subtilis* 62305 using broth dilution assay. " – " means 200 μL of bacterial solution as negative control; " + " means 200 μL untreated media as positive control; " **A** ": *M. smegmatis* MC[2] 155; " **B** ": *B. subtilis* 62305. *RIF*: rifampicin, *AMP*: ampicillin.

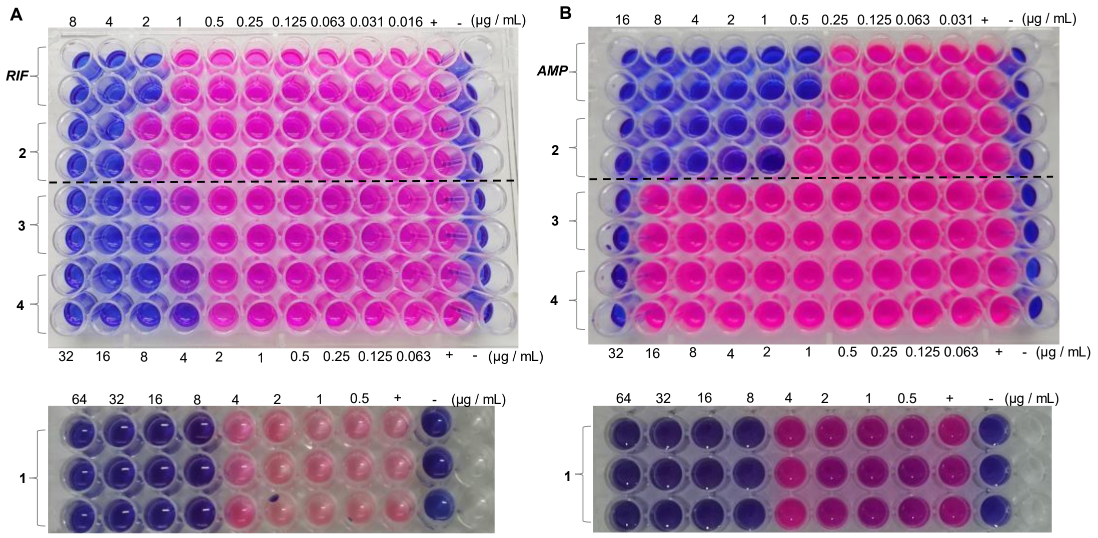

Figure S6 The cytotoxicity of compound **1** against NCM-460 cells and 4T-1 cells by the MTT assay.

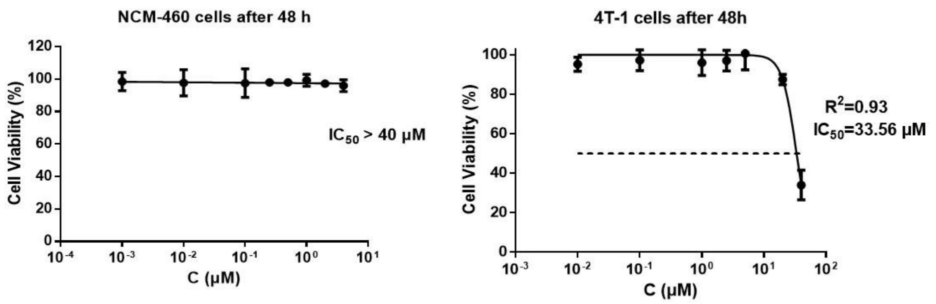

Figure S7 The HMRS and MS/MS of compound **1.**

The HRMS of compound **1**

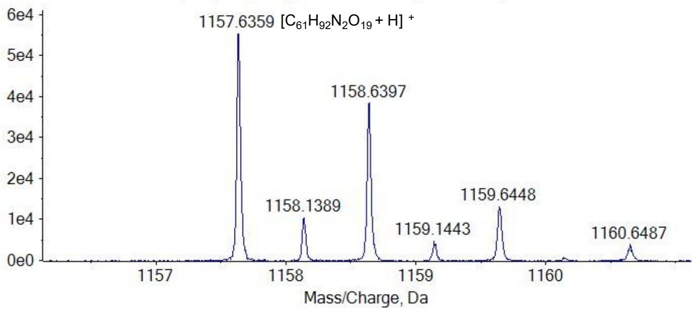

#	Analyte Peak Name	Accuracy	Ion Ratio	Formula	Precursor Mass	Accuracy Acceptance	Concentration Acceptance	Found At Mass	Mass Error (ppm)	Library Hit	Library Score	Isotope Ratio Difference
1	ShiJie01+H	N/A	N/A	C61H92N2O19	1157.637	Pass	Pass	1157.6359	-0.7	No data for Library Hit	N/A	3.2
2	ShiJie01+Na	N/A	N/A	C61H92N2O19	1179.619	Pass	Pass	1179.6178	-0.7	No data for Library Hit	N/A	3.0

The MS/MS of compound **1**

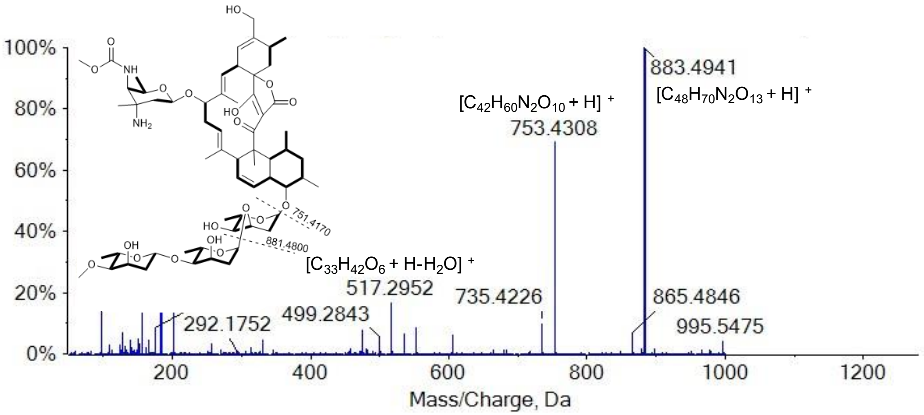

Figure S8 The MS/MS of compound **2.**

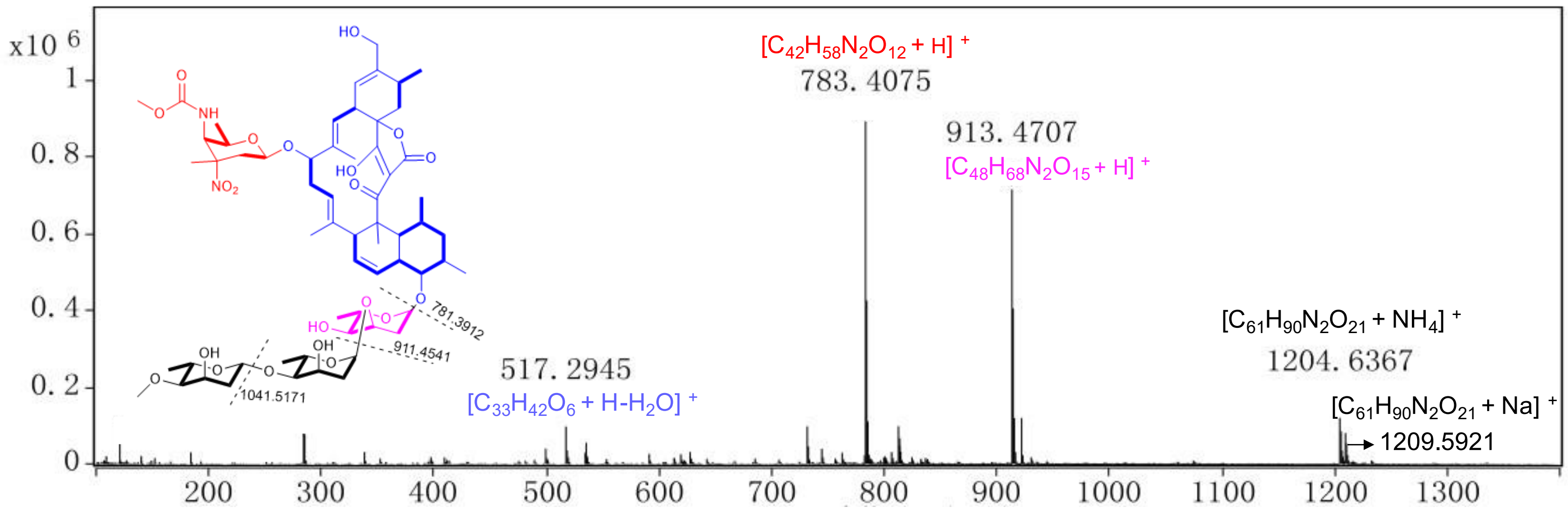

Figure S9 The HRMS of compound **3.**

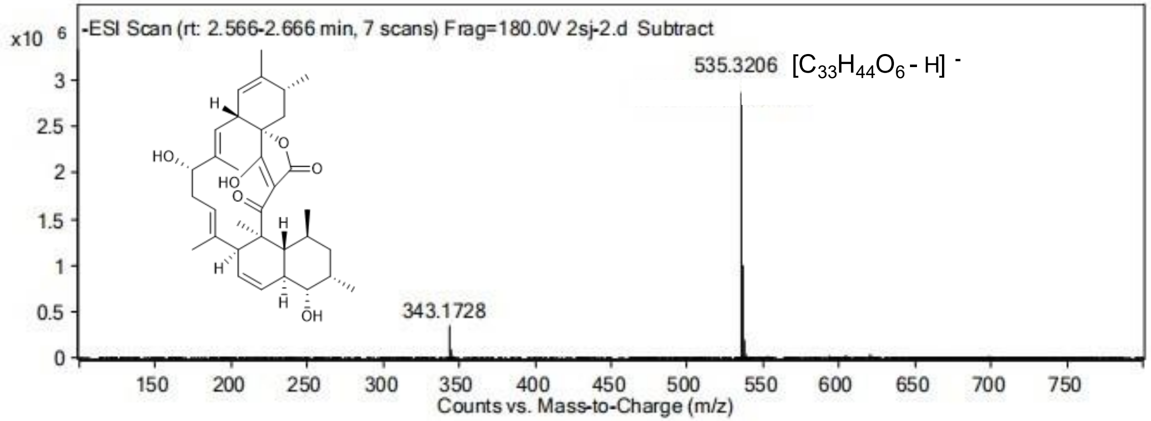

Figure S10 The HRMS of compound **4.**

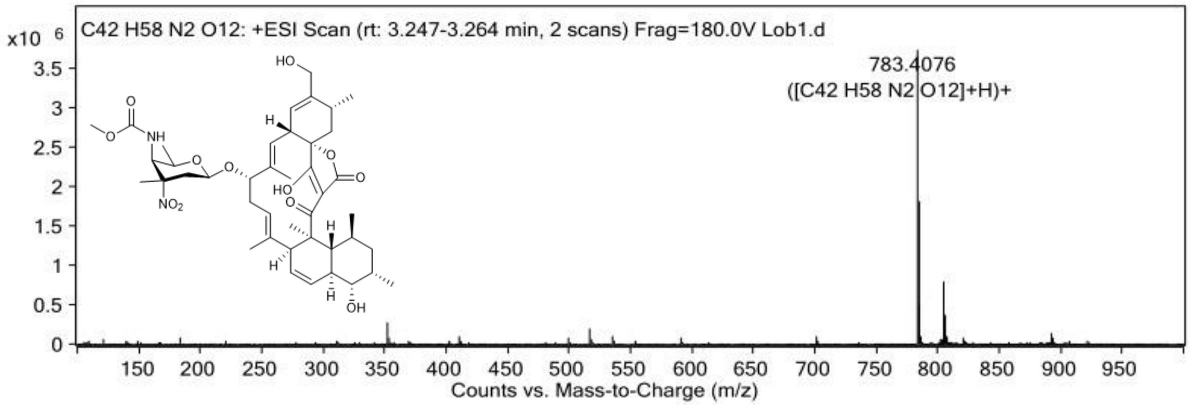

Figure S11 ^{1}H-NMR spectrum of **1** in CD_3OD (400 MHz).

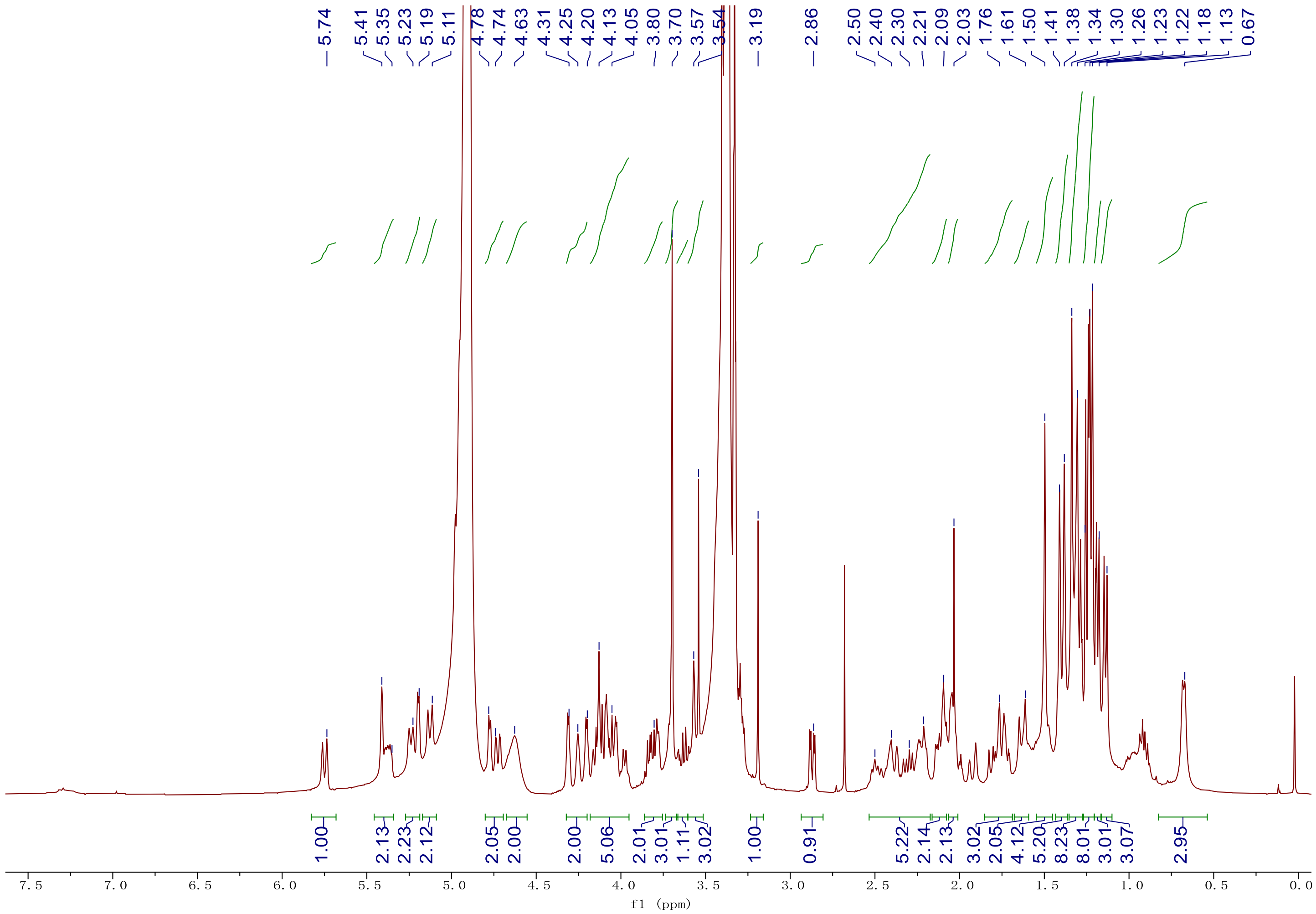

Figure S12 ^{13}C NMR spectrum of **1** in CD_3OD (100 MHz).

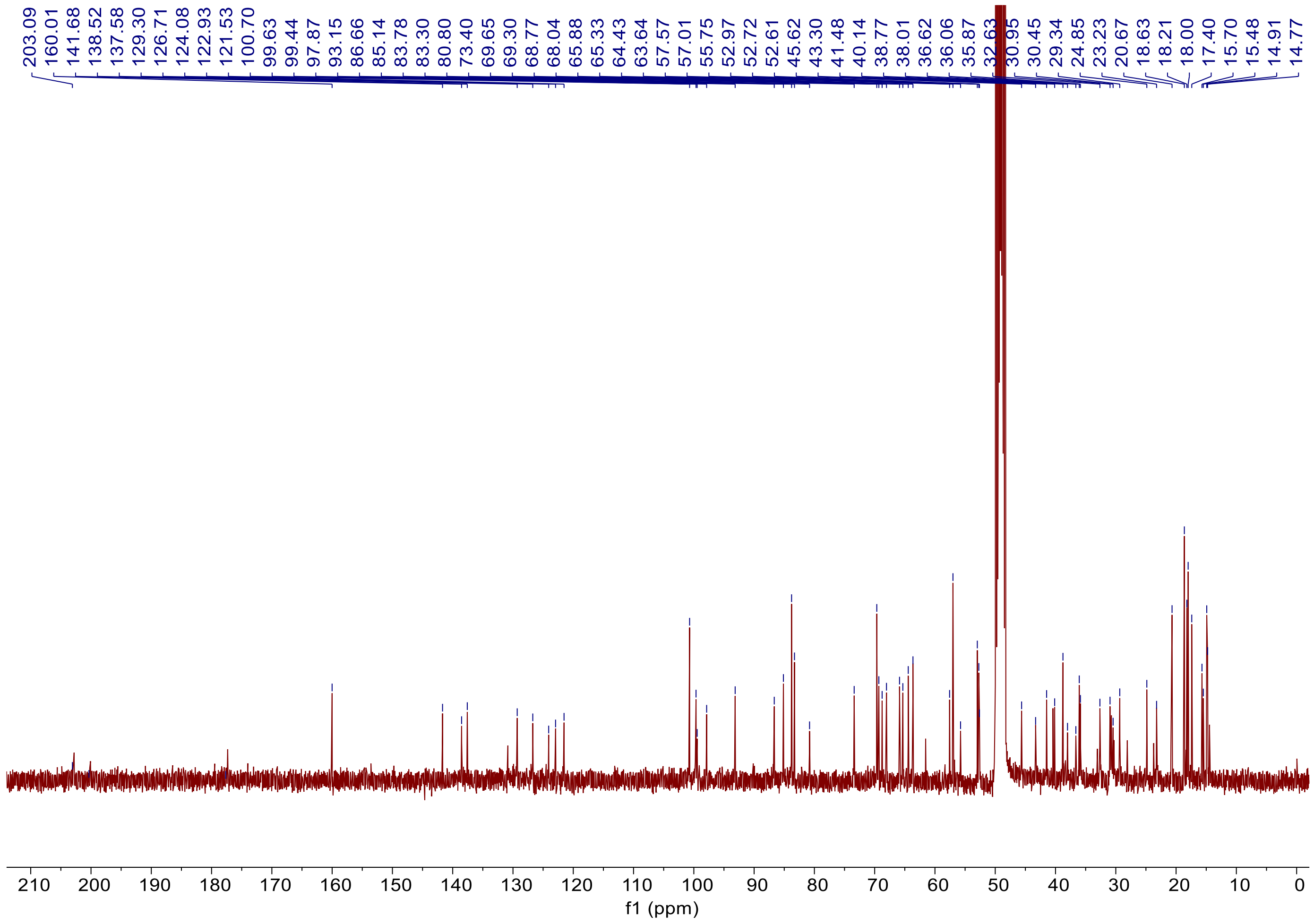

Figure S13 ^{1}H NMR spectrum of **2** in CD_3OD (600 MHz).

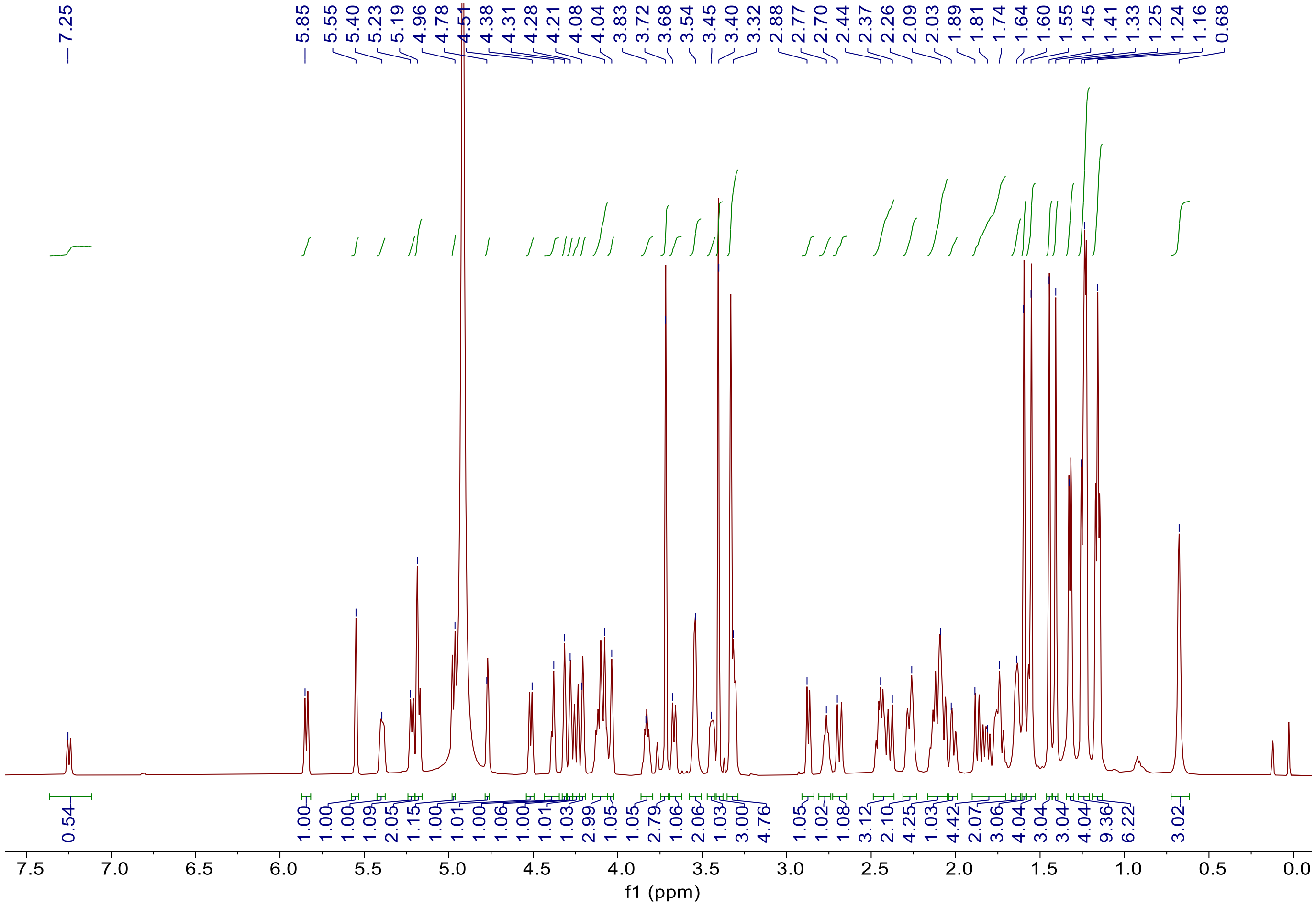

Figure S14 ^{13}C NMR spectrum of **2** in CD_3OD (150 MHz).

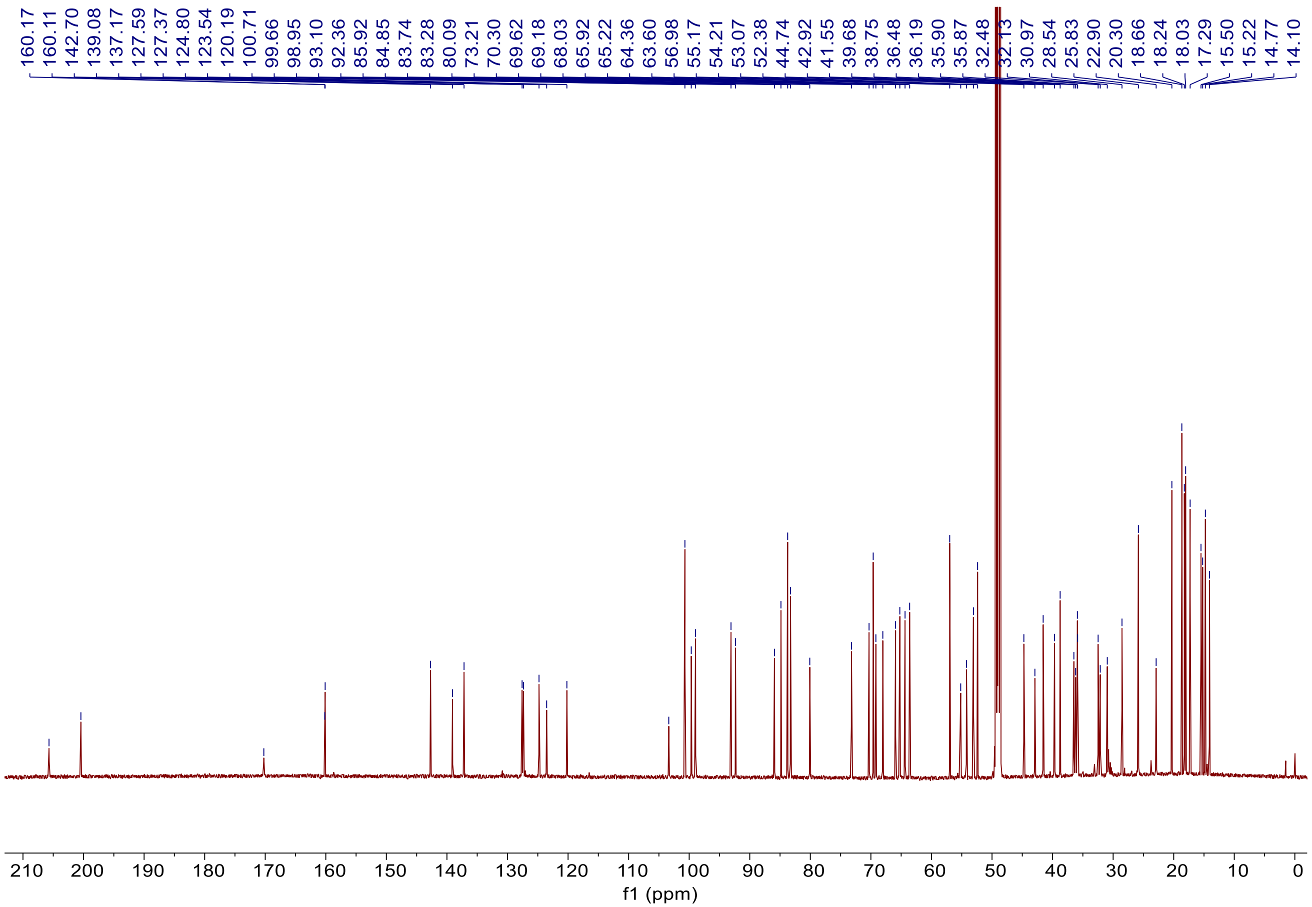

Figure S15 DEPT 90 spectrum of **2** in CD_3OD.

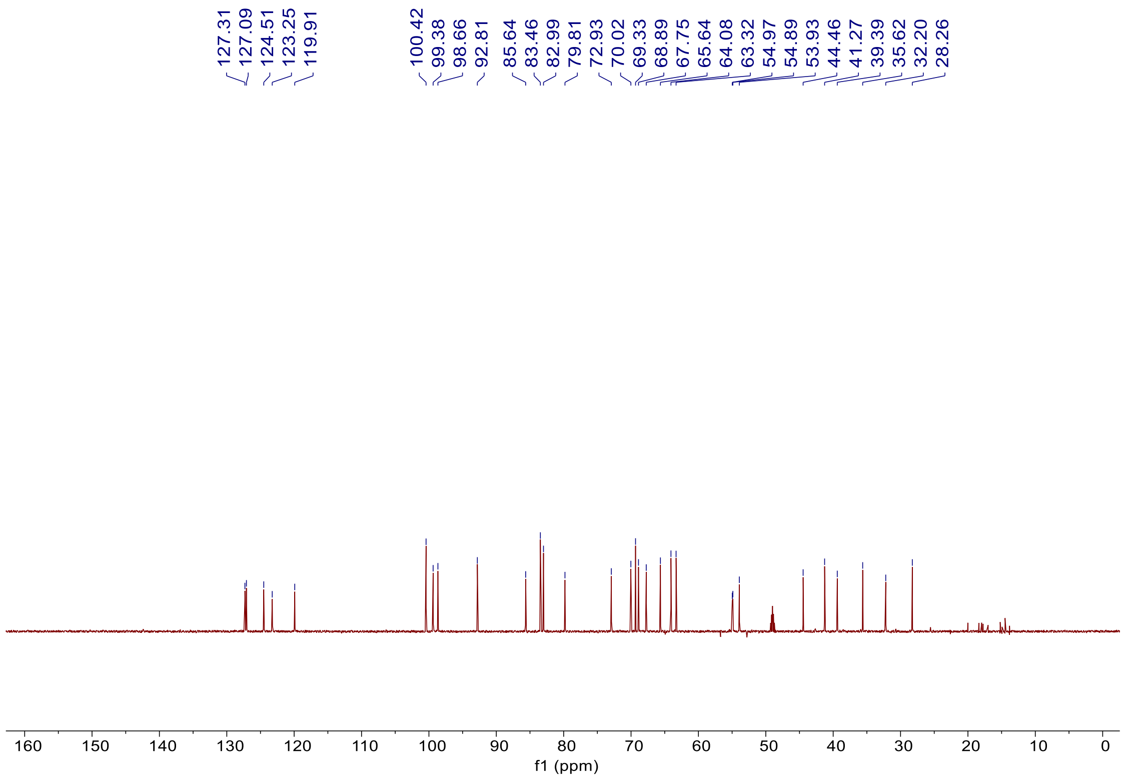

Figure S16 DEPT 135 spectrum of **2** in CD_3OD.

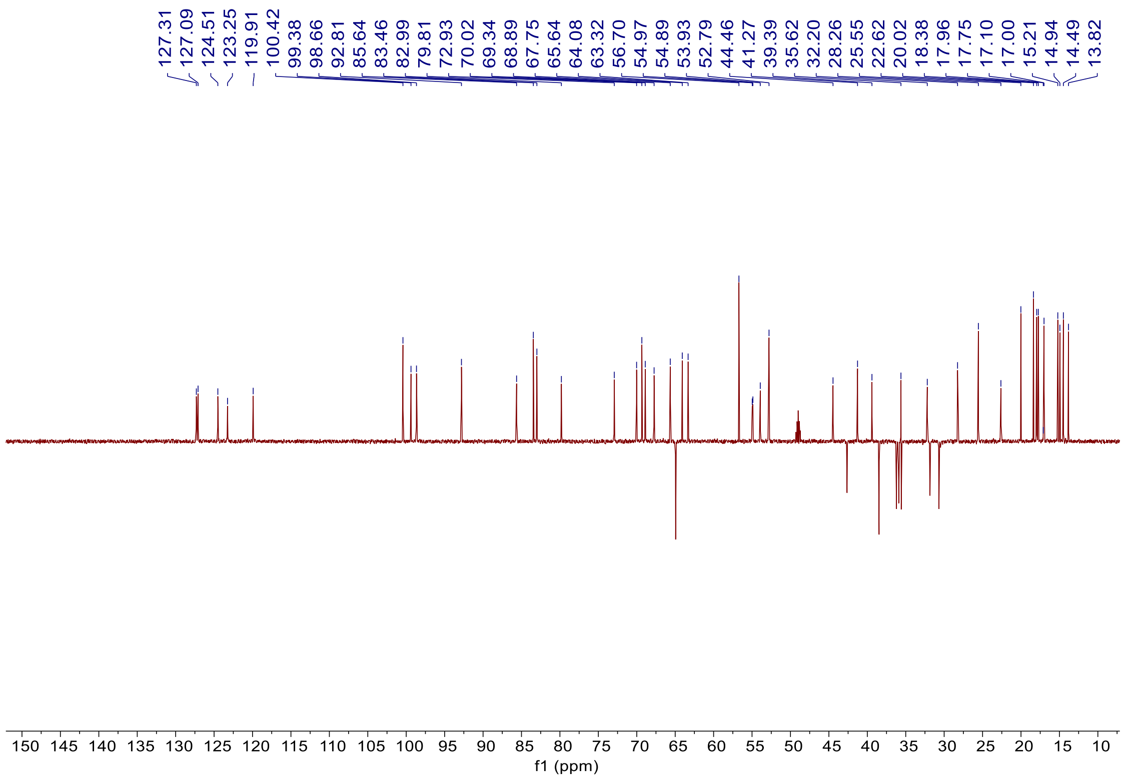

Figure S17 ^{1}H-^{1}H COSY spectrum of **2** in CD_3OD.

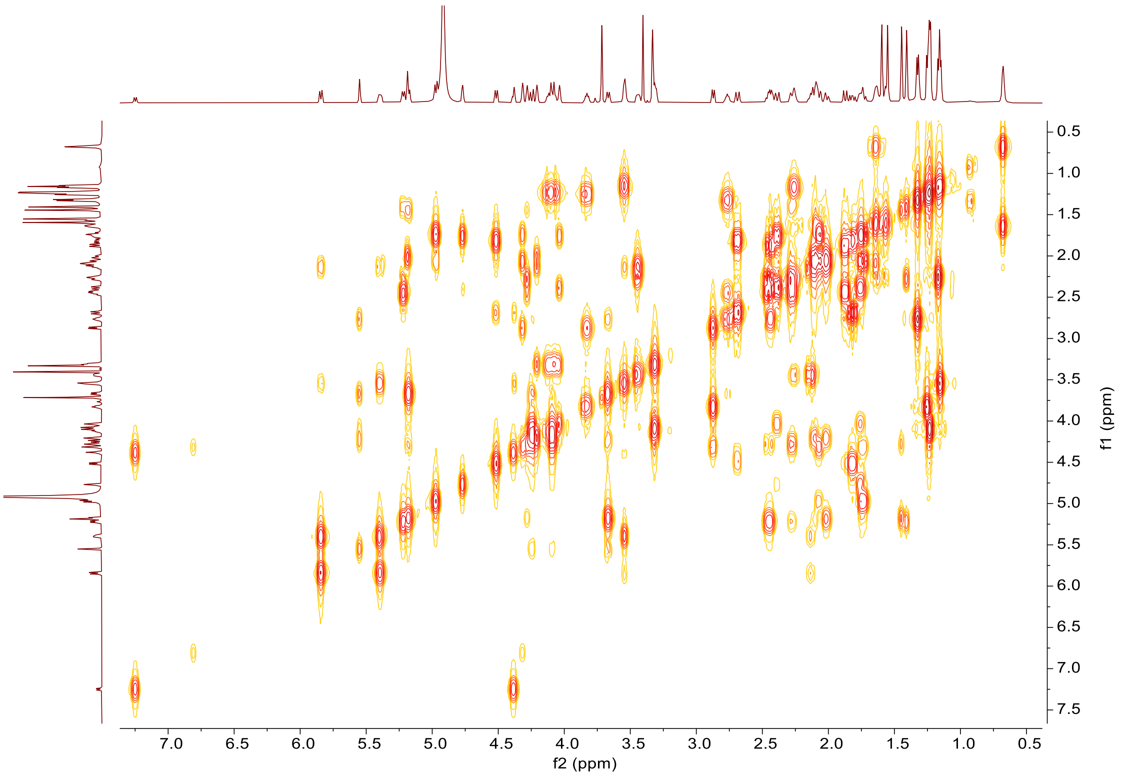

Figure S18 HSQC spectrum of **2** in CD_3OD.

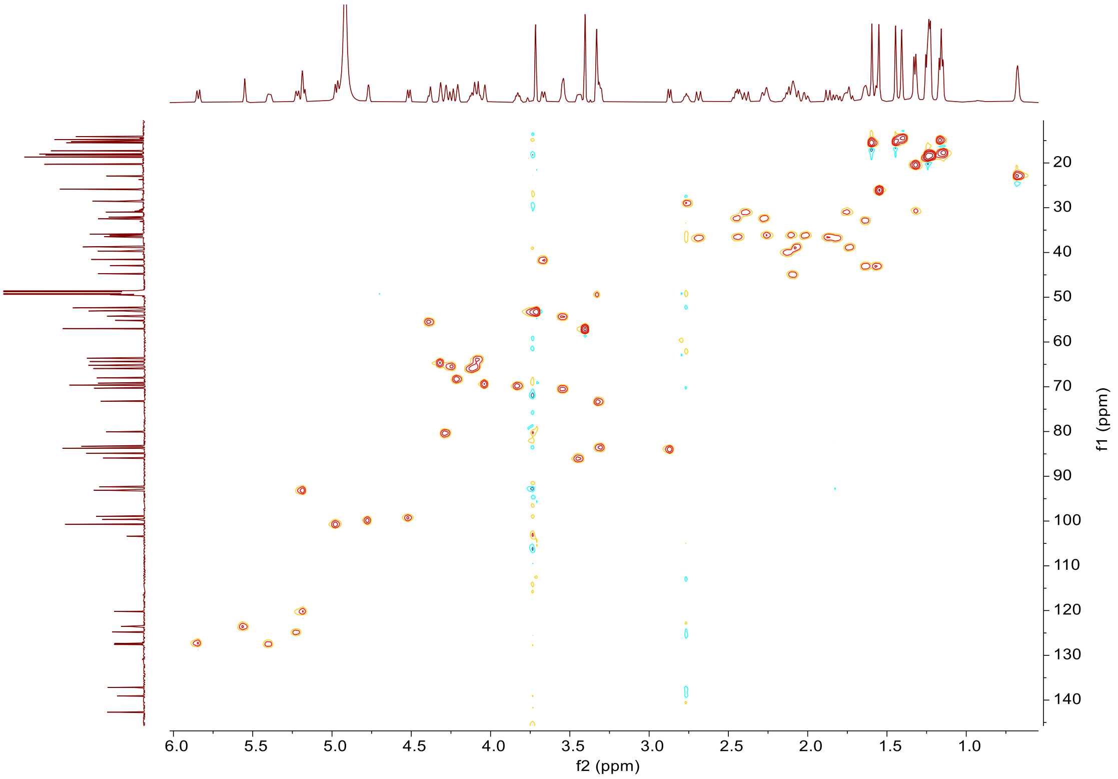

Figure S19 HMBC spectrum of **2** in CD_3OD.

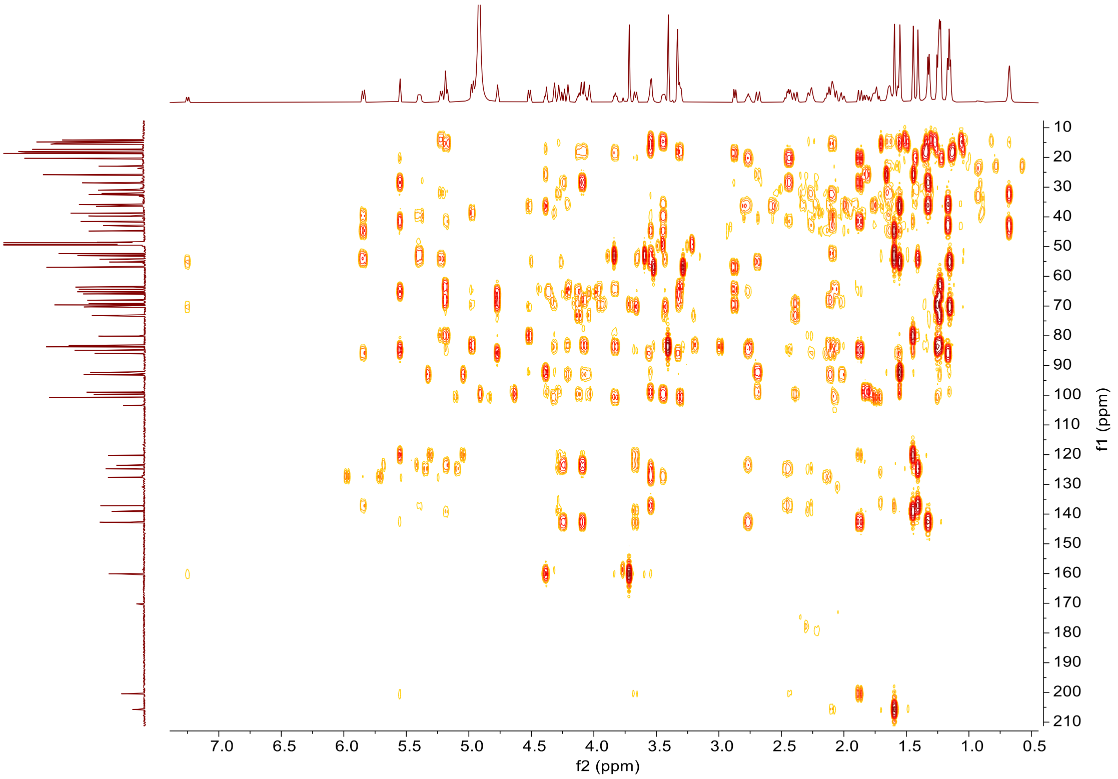

Figure S20 NOESY spectrum of **2** in CD_3OD.

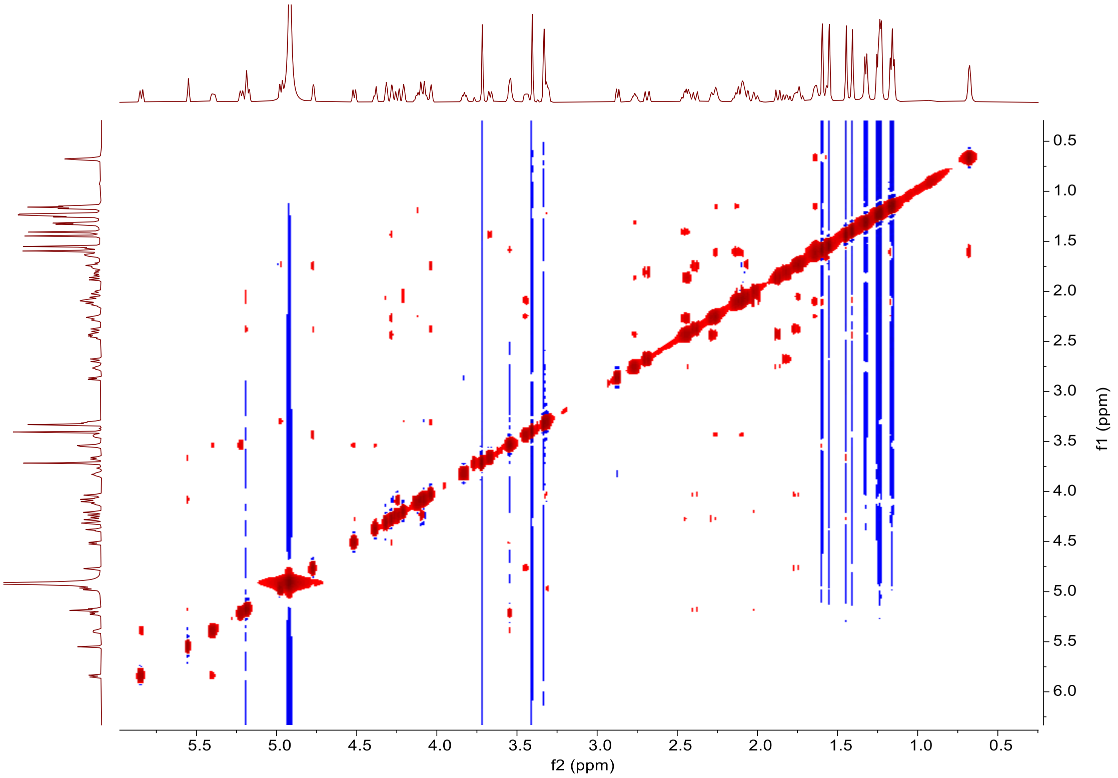

Figure S21 ^{1}H NMR spectrum of **3** in CD_3OD (500 MHz).

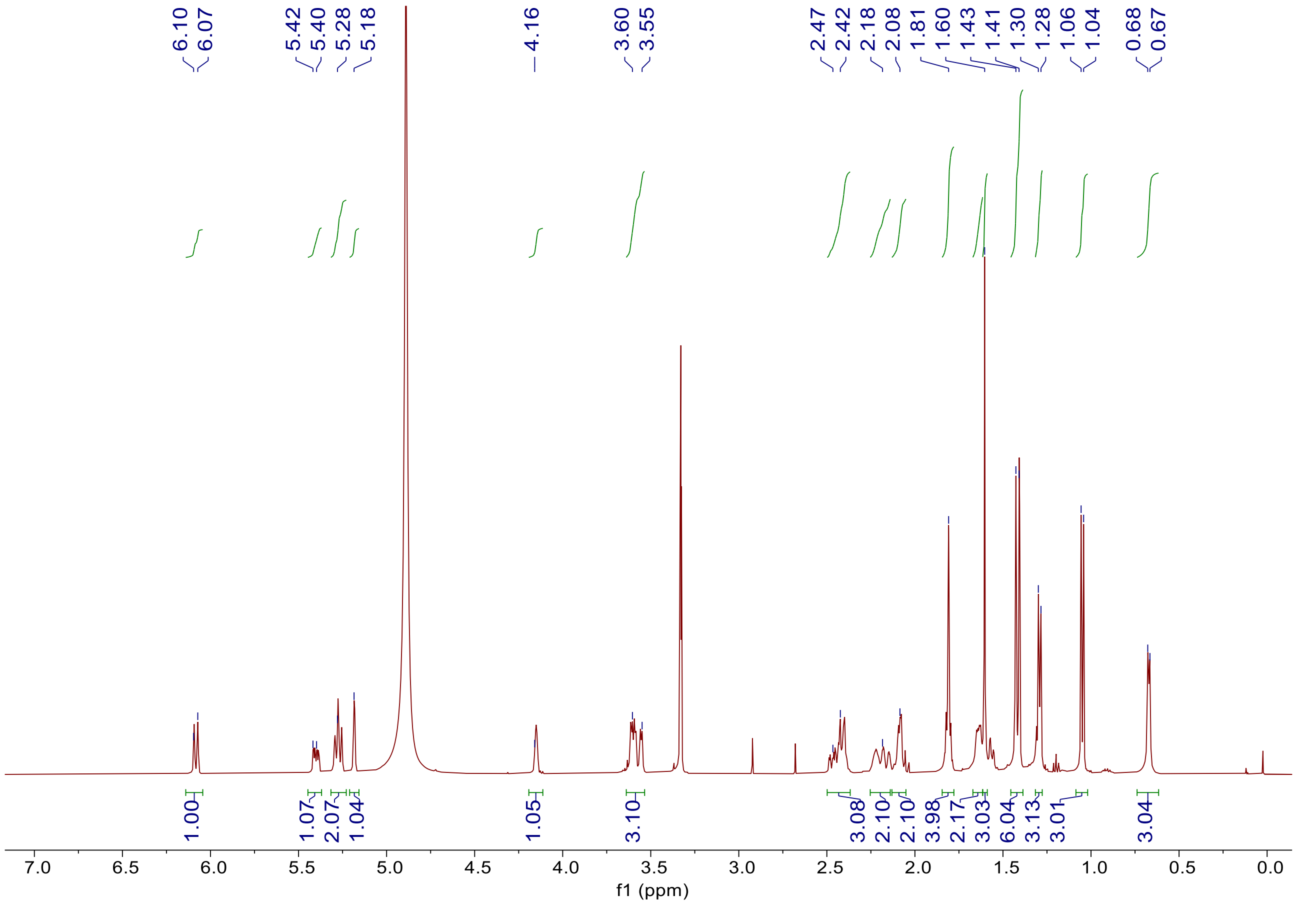

Figure S22 ^{13}C-NMR spectrum of **3** in CD_3OD (125 MHz).

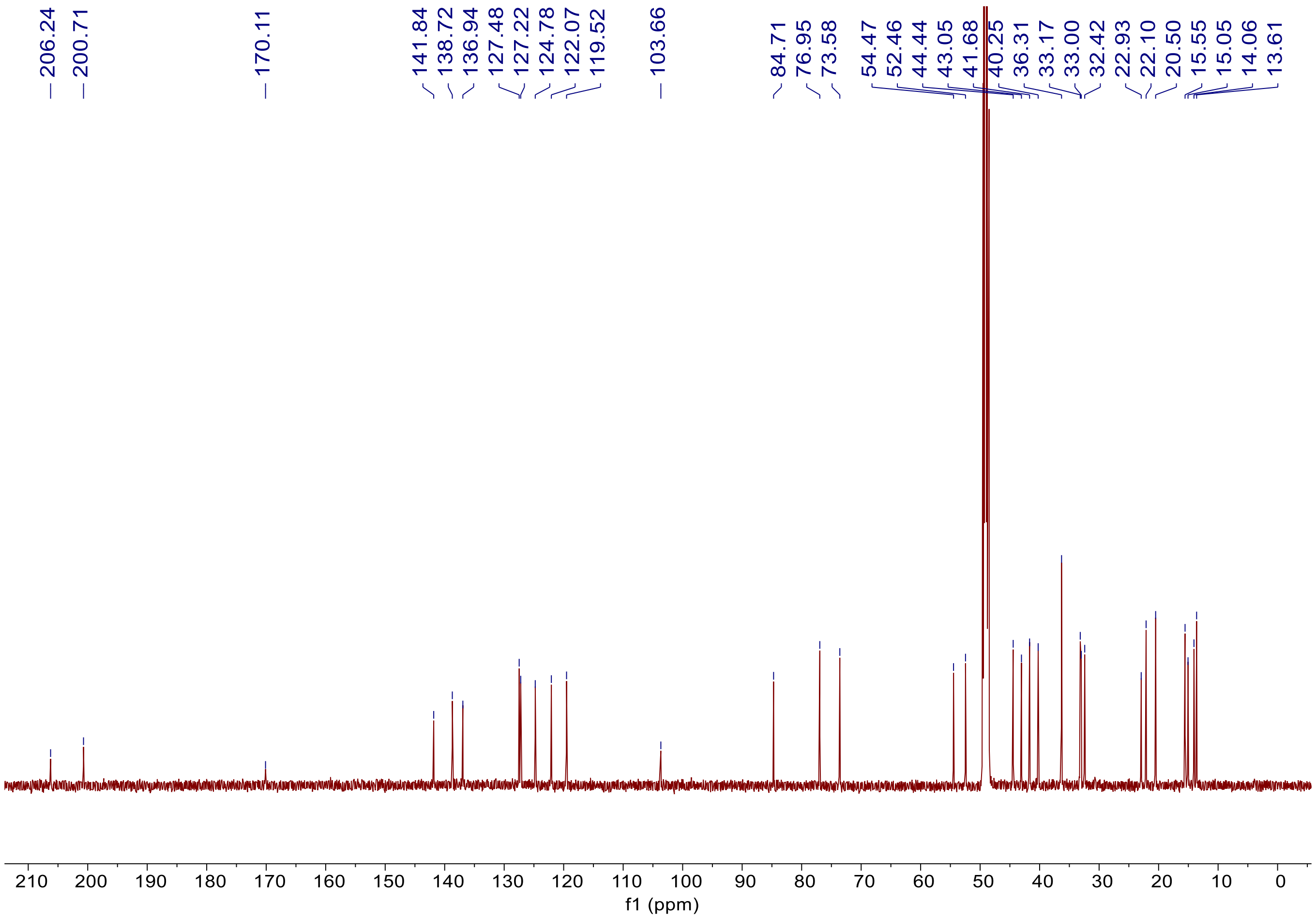

Figure S23 DEPT 90 spectrum of **3** in CD_3OD.

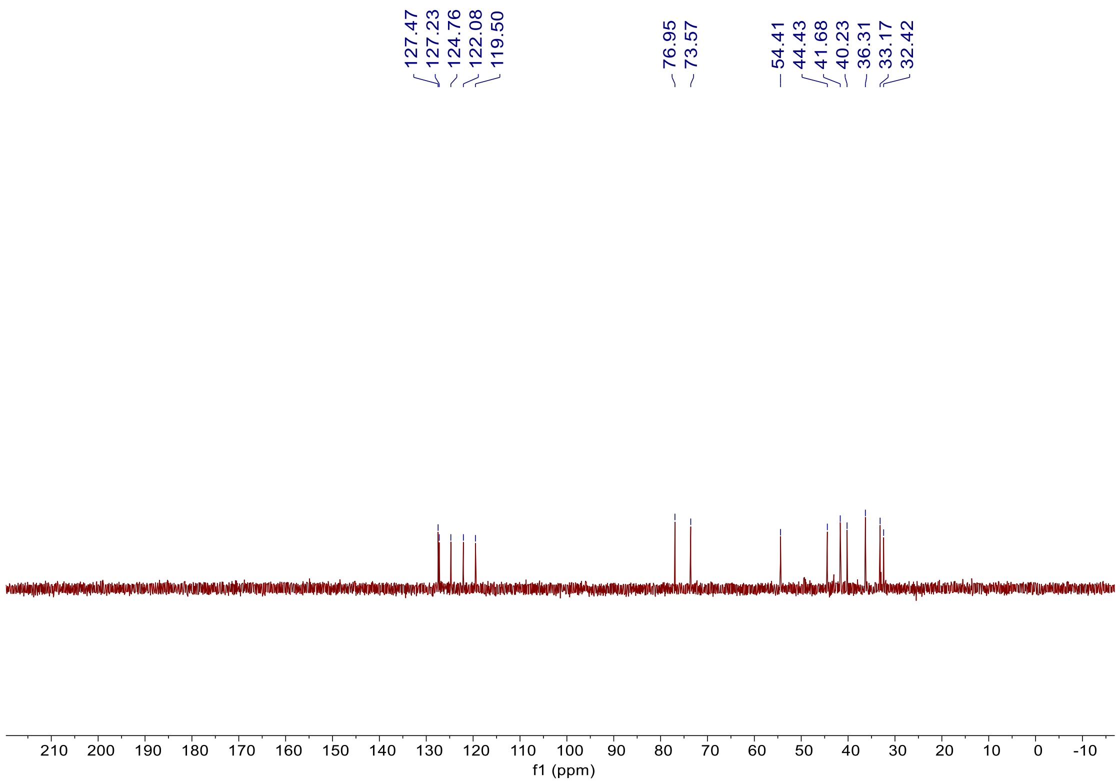

Figure S24 DEPT 135 spectrum of **3** in CD_3OD.

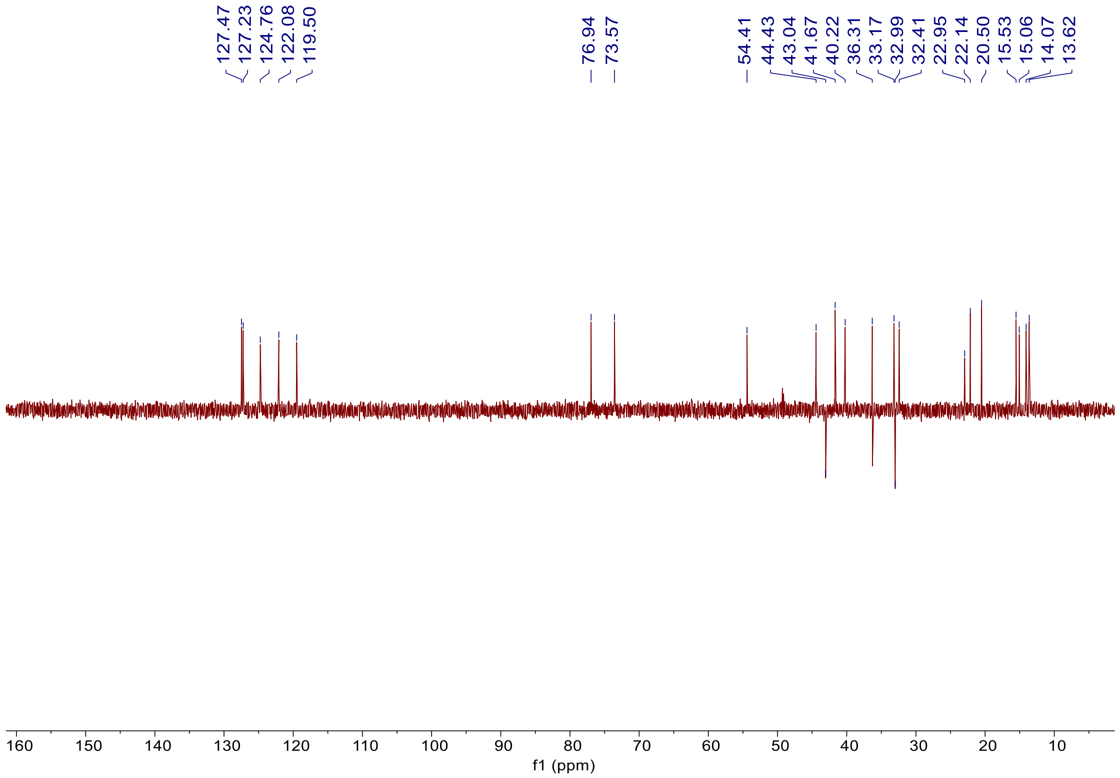

Figure S25 ^{1}H-^{1}H COSY spectrum of **3** in CD_3OD.

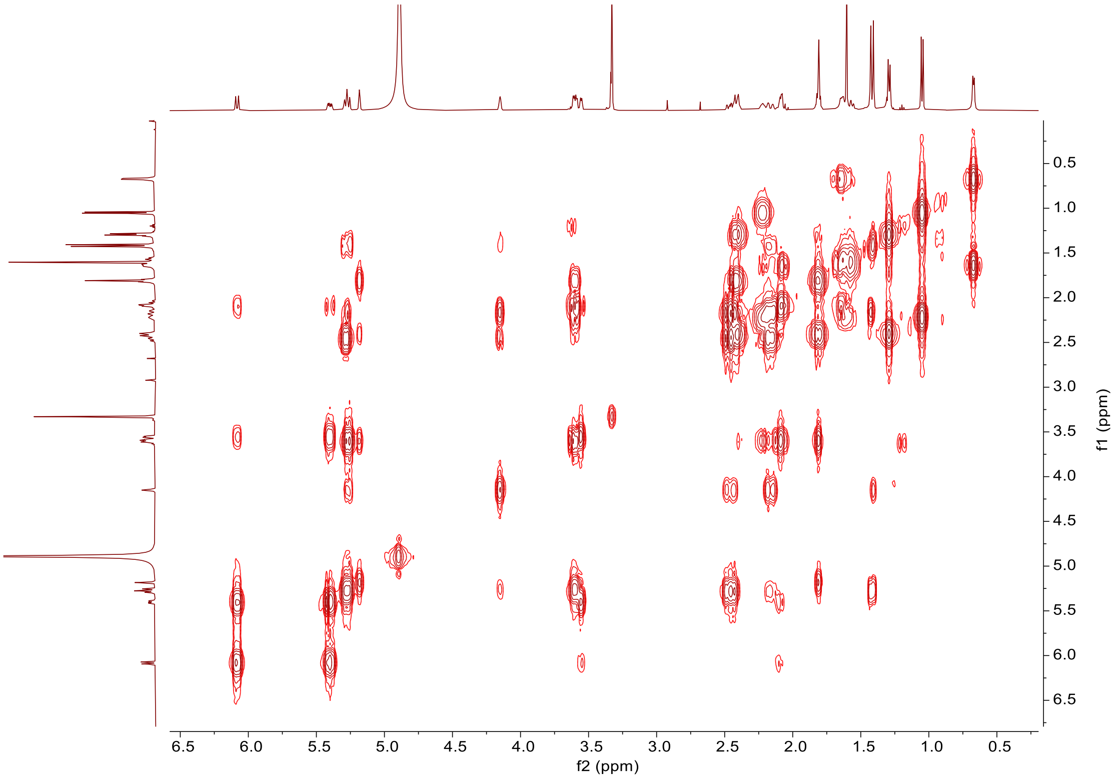

Figure S26 HSQC spectrum of **3** in CD_3OD.

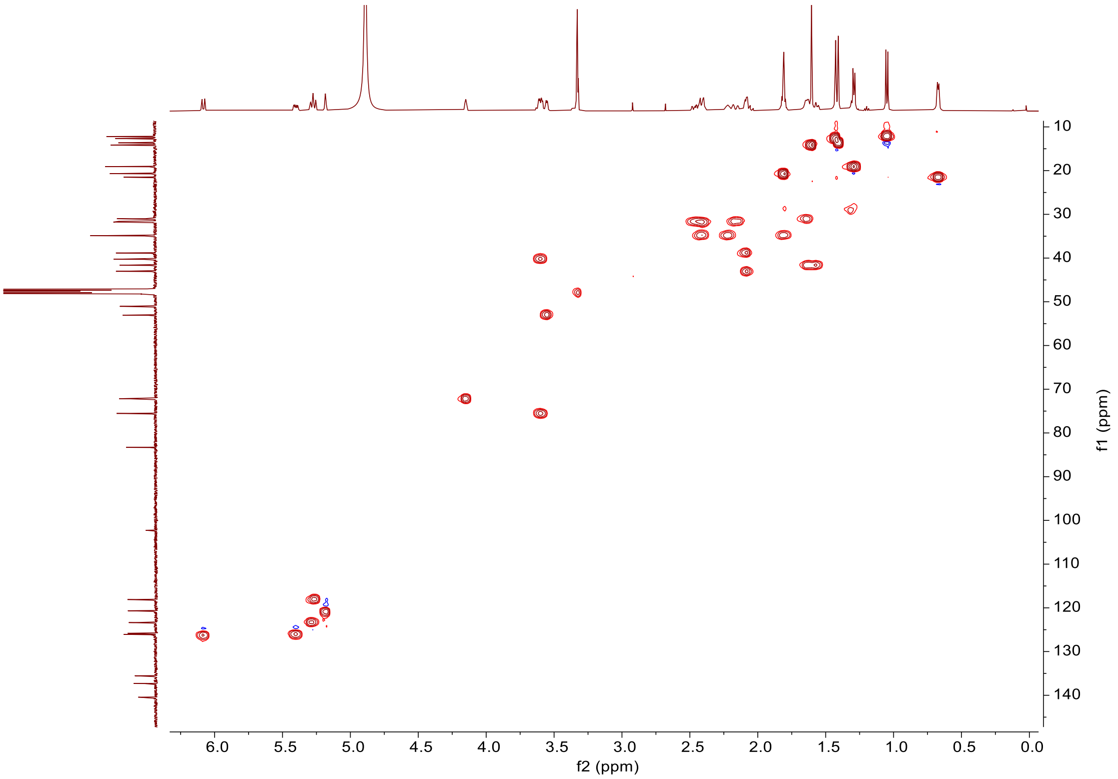

Figure S27 HMBC spectrum of **3** in CD_3OD.

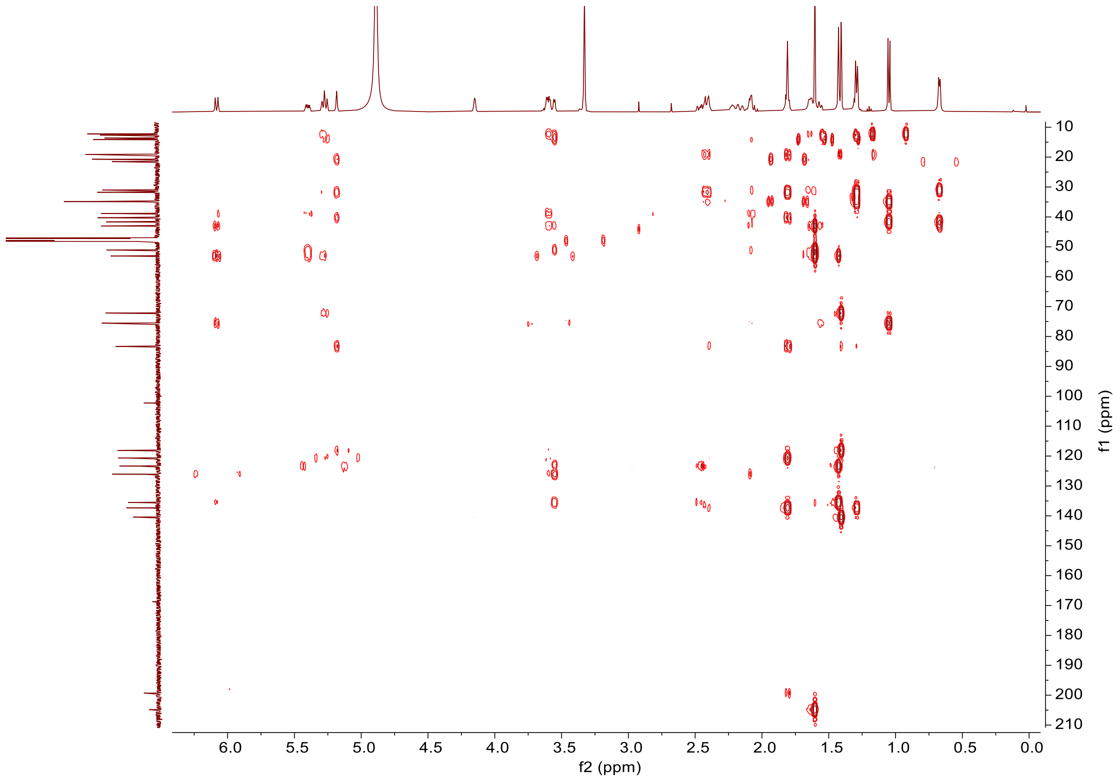

Figure S28 NOESY spectrum of **3** in CD_3OD.

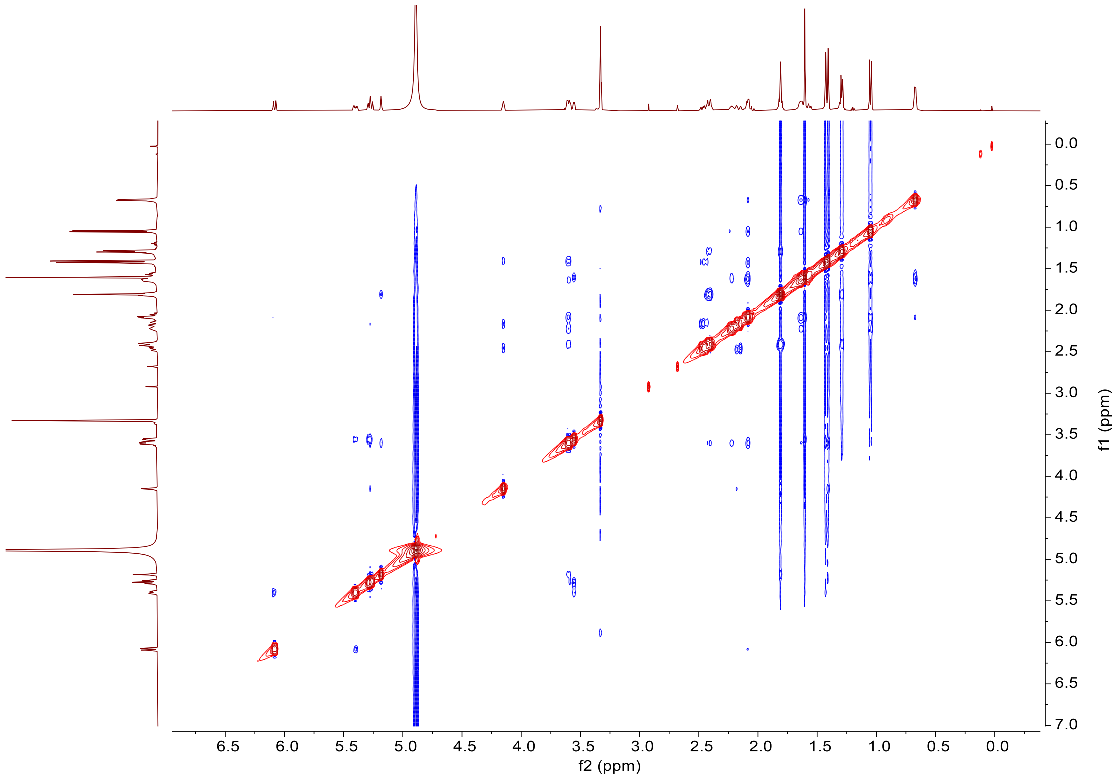

Figure S29 ^{1}H NMR spectrum of **4** in CD_3OD (600 MHz).

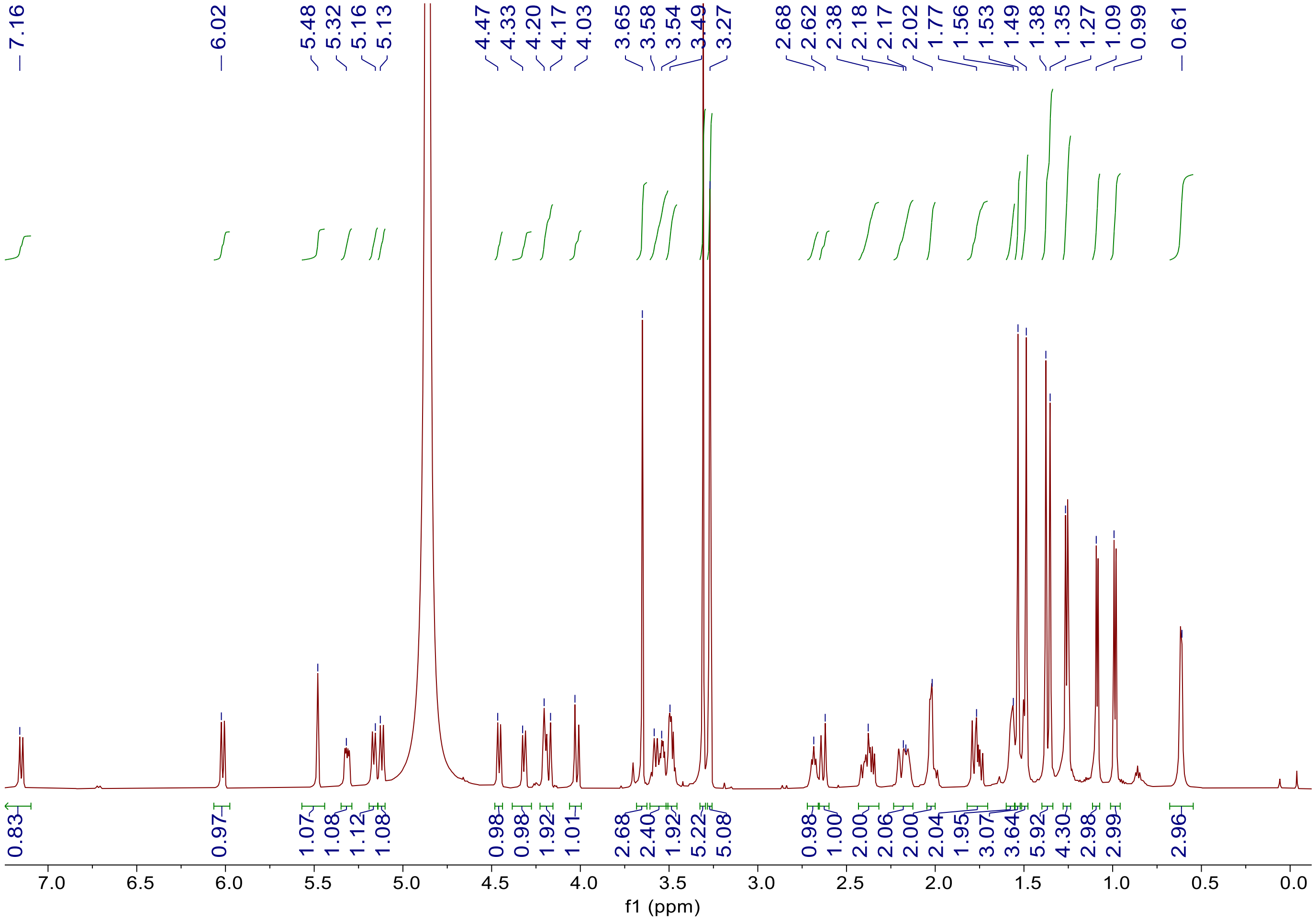

Figure S30 ^{13}C NMR spectrum of **4** in CD_3OD (150 MHz).

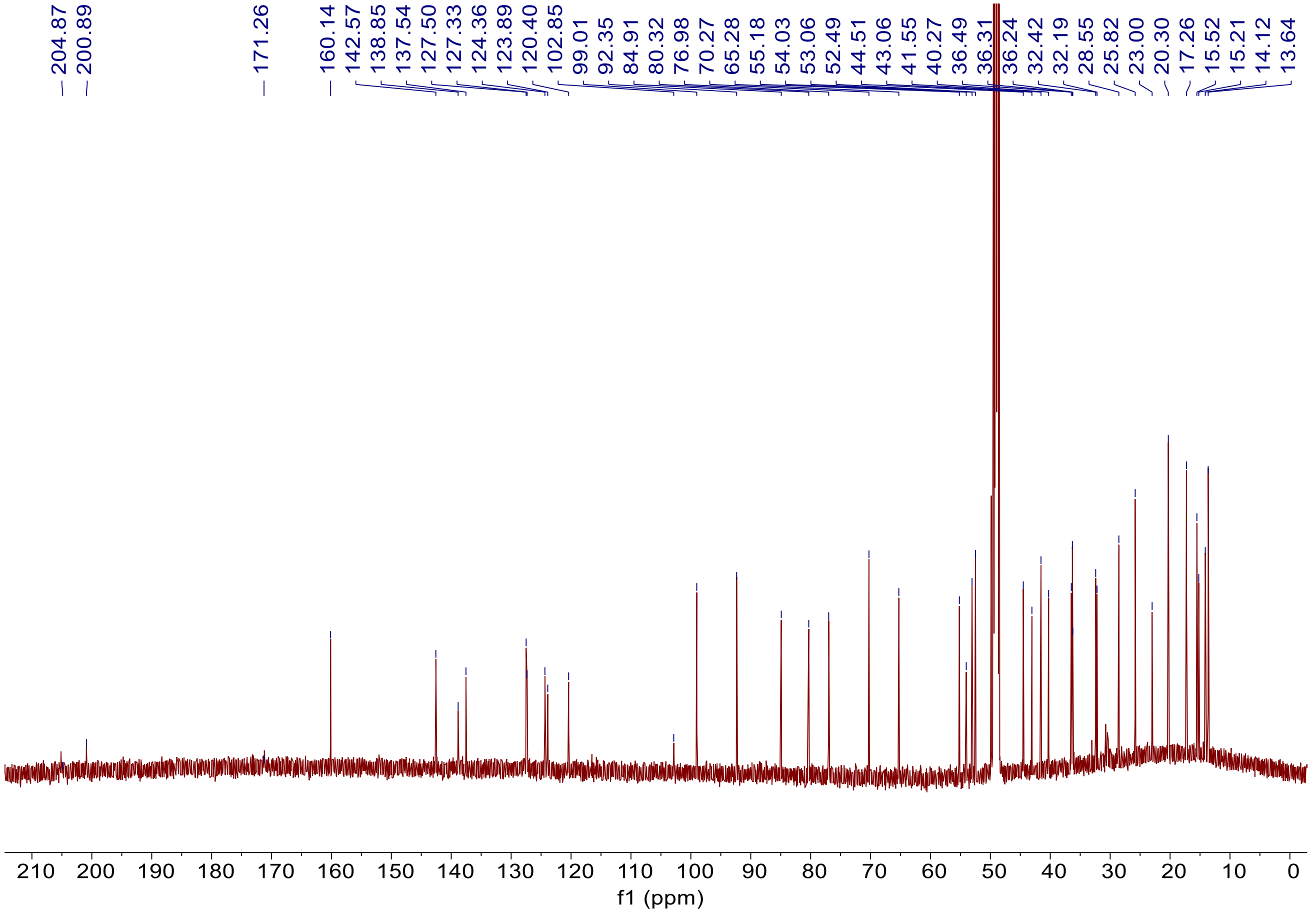

Figure S31 DEPT 90 spectrum of **4** in CD_3OD.

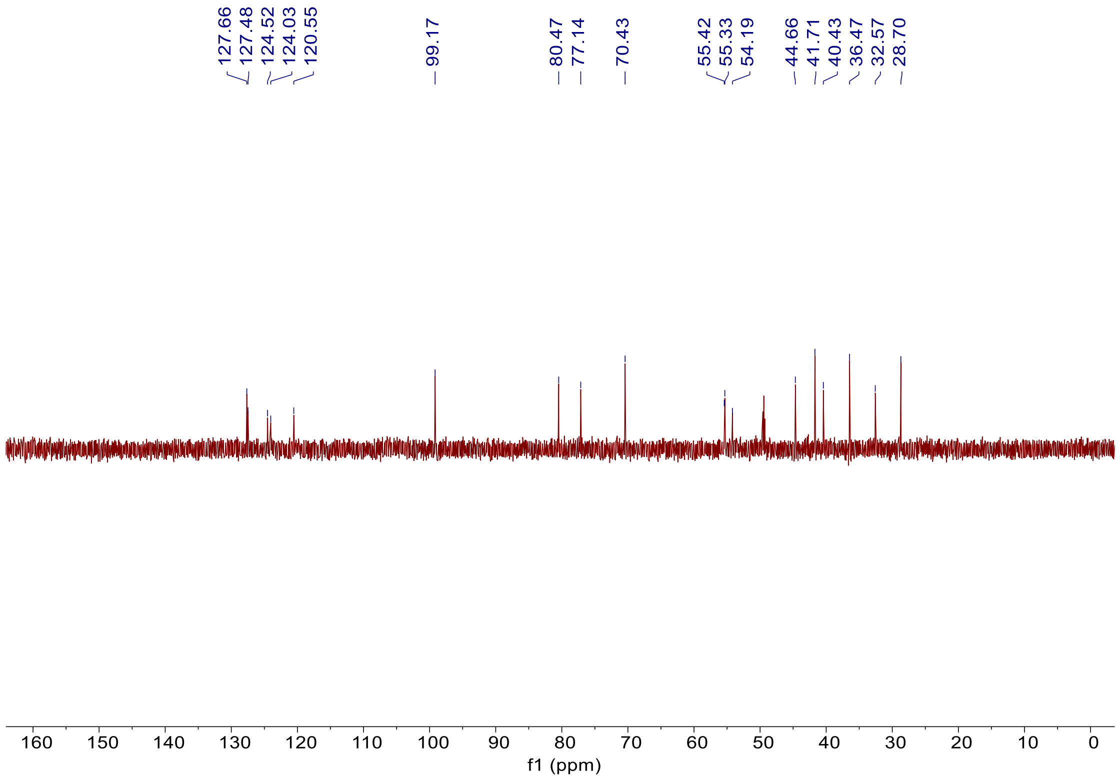

Figure S32 DEPT 135 spectrum of **4** in CD_3OD.

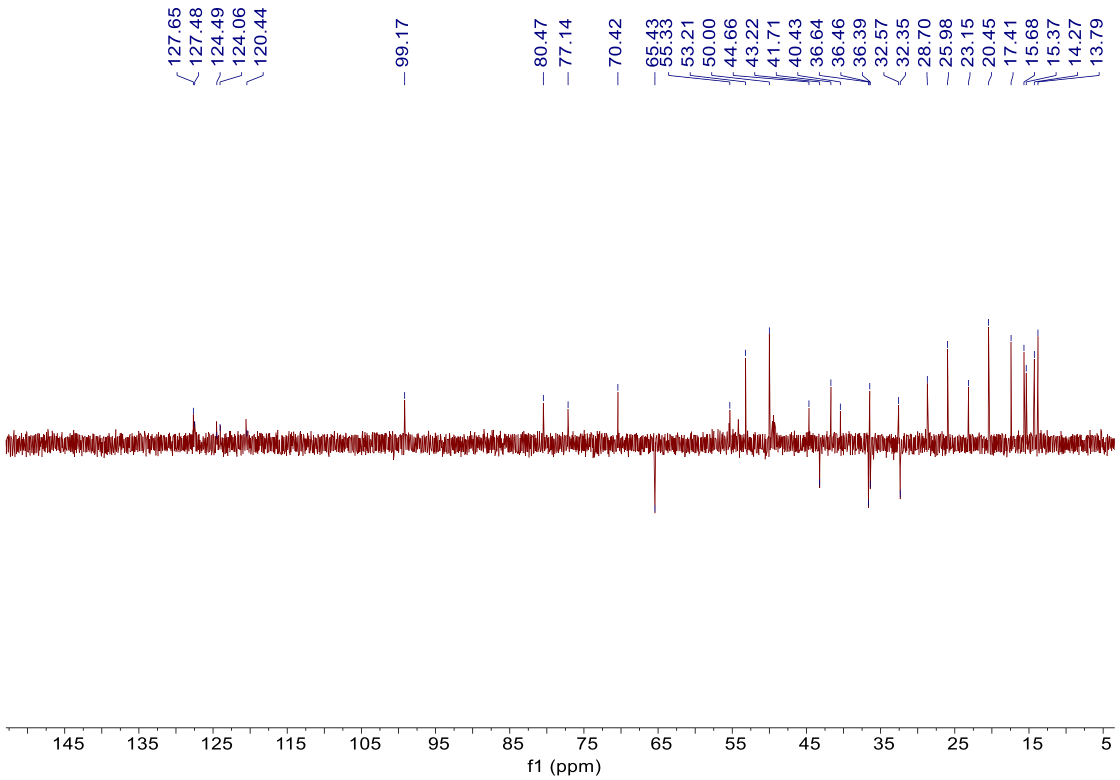

Figure S33 ^{1}H-^{1}H COSY spectrum of **4** in CD_3OD.

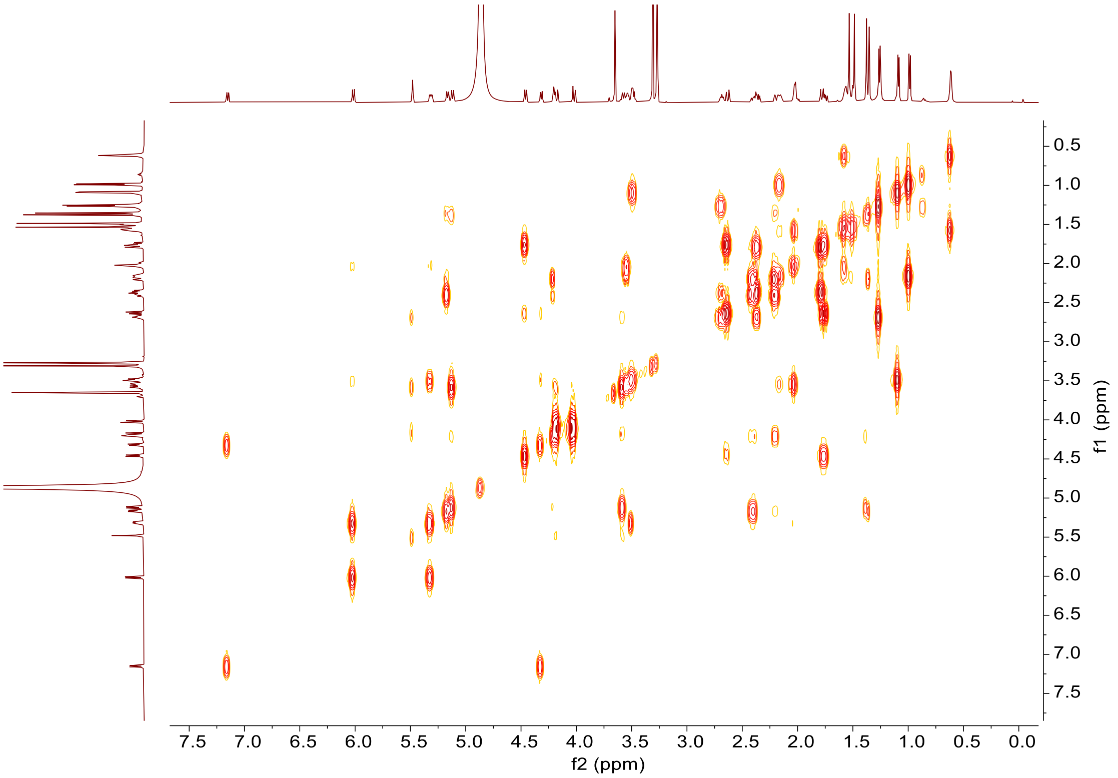

Figure S34 HSQC spectrum of **4** in CD_3OD.

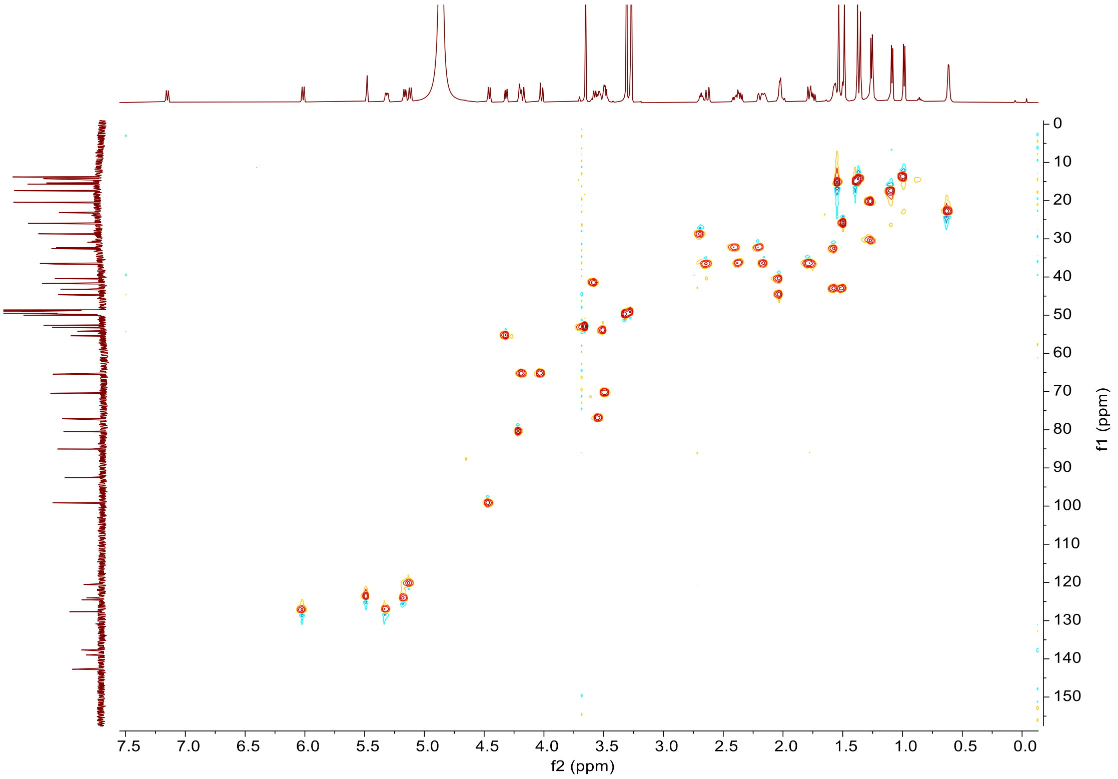

Figure S35 HMBC spectrum of **4** in CD_3OD.

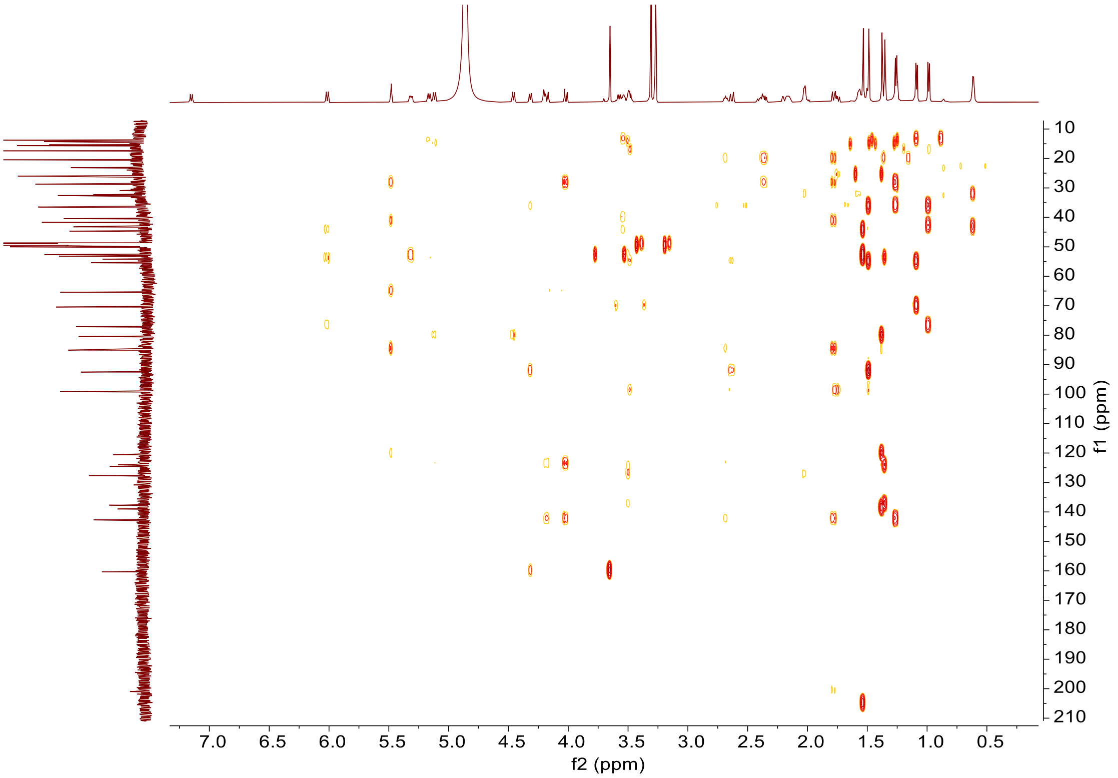

Figure S36 NOESY spectrum of **4** in CD_3OD.

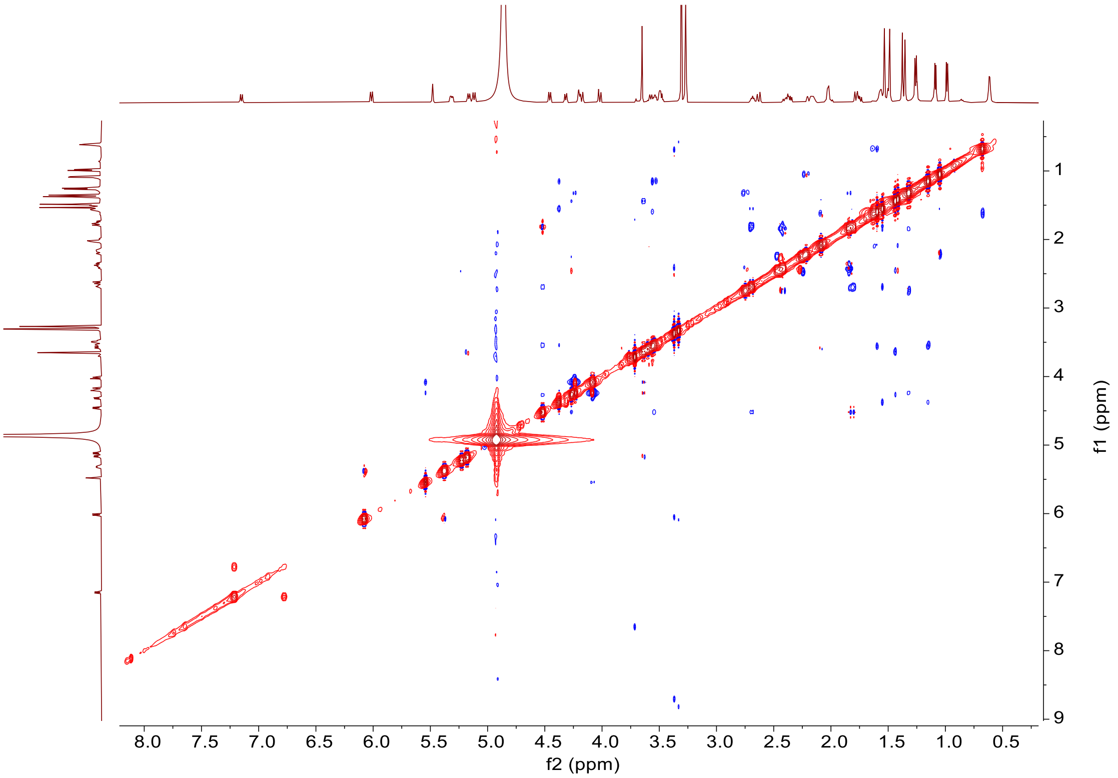

References

[1] Jiang, Z.D.; Jensen, P.R.; Fenical, W. Lobophorins A and B, new antiinflammatory macrolides produced by a tropical marine bacterium. *Bioorg. Med. Chem. Lett.* 1999, 9, 2003-2006.

[2] Chen, C.; Wang, J.; Guo, H.; Hou, W.; Yang, N.; Ren, B.; Liu, M.; Dai, H.; Liu, X.; Song, F.; Zhang, L. Three antimycobacterial metabolites identified from a marine-derived *Streptomyces* sp. MS100061. *Appl. Microbiol. Biotechnol.* 2013, 97 (9), 3885-3892.

[3] Mallams, A. K., Puar, M. S., Rossman, R. R., McPhail, A. T., Macfarlane, R. D., Stephens, R. L. Kijanimicin. Part 3. Structure and absolute stereochemistry of kijanimicin. *J. Chem. Soc*. 1983, 1, 1497-1534.

[4] Luo, M. H.; Tang, L. J.; Dong, Y. L.; Huang, H.; Deng, Z.; Sun, Y. Antibacterial natural products lobophorin L and M from the marine-derived *Streptomyces* sp. 4506. *Nat. Prod. Res*. 2020, 27, 5581-5587.

[5] Tan B, Chen S, Zhang Q, et al. Heterologous expression leads to discovery of diversified lobophorin analogues and a flexible glycosyltransferase. *Org. Lett*., 2020.

[6] Wei, R. B.; Xi, T.; Li, J.; Wang, P.; Li, F.-C.; Lin, Y.-C.; Qin, S., Lobophorin C and D, new kijanimicin derivatives from a marine sponge-associated actinomycetal strain AZS17. *Mar. Drugs* 2011, 9 (3), 359-368.

[7] Niu, S.; Li, S.; Chen, Y.; Tian, X.; Zhang, H.; Zhang, G.; Zhang, W.; Yang, X.; Zhang, S.; Ju, J.; Zhang, C. Lobophorins E and F, new spirotetronate antibiotics from a South China Sea-derived *Streptomyces* sp SCSIO 01127. *J. Antibiot.* 2011, 64 (11), 711-716.

[8] Pan, H. Q.; Zhang, S. Y.; Wang, N.; Li, Z. L.; Hua, H. M.; Hu, J. C.; Wang, S. J. New spirotetronate antibiotics, lobophorins H and I, from a South China Sea-derived *Streptomyces* sp. 12A35. *Mar. Drugs* 2013, 11 (10), 3891-901.

[9] Song C F; Pan H Q; C, H. J., Isolation and identification of a new antibiotic, lobophorin J, from a deep sea-derived *Streptomyces* sp. 12A35. *Chin. J. Antibiot* 2015, 40 (10), 721-727.

[10] Brana, A. F.; Sarmiento-Vizcaino, A.; Osset, M.; Perez-Victoria, I.; Martin, J.; de Pedro, N.; de la Cruz, M.; Diaz, C.; Vicente, F.; Reyes, F.; Garcia, L. A.; Blanco, G., Lobophorin K, a new natural product with cytotoxic activity produced by *Streptomyces* sp. M-207 associated with the deep-sea coral *Lophelia pertusa*. *Mar. Drugs* 2017, 15 (5), 144.

[11] Cruz, P. G.; Fribley, A. M.; Miller, J. R.; Larsen, M. J.; Schultz, P. J.; Jacob, R. T.; Tamayo-Castillo, G.; Kaufman, R. J.; Sherman, D. H., Novel lobophorins inhibit oral cancer cell growth and induce Atf4- and Chop-dependent cell death in murine fibroblasts. *ACS Med. Chem. Lett.* 2015, 6 (8), 877-81.

[12] Zhang, C.; Ding, W.; Qin, X.; Ju, J., Genome sequencing of *Streptomyces olivaceus* SCSIO T05 and activated production of lobophorin CR4 via metabolic engineering and genome mining. *Mar. Drugs* 2019, 17 (10), 593.

[13] Tan, B.; Zhang, Q.B.; Xiao, J.; Yuan, C.S.; Chen, Y.C.; Chen, S.Q.; Zhang, W.M.; Zhu, Y.G.; Zhang, C.S. Characterization of the P450 monooxygenase LobP1 as C-32 hydroxylase in Lobophorin biosynthesis. *Chin. J. Chem*. 2023, DOI:

10.1002/cjoc.202200818.
